# Supplementary material for: APOE4 impacts cortical neurodevelopment and alters network formation in human brain organoids
Source: Stem Cell Reports. 2025 Jun 19;20(7):102537. doi: 10.1016/j.stemcr.2025.102537 (PMC12277819; doi:10.1016/j.stemcr.2025.102537)
Supplement: Document S1. Figures S1–S6, Tables S1–S3, and supplemental methods [file mmc1.pdf]

Stem Cell Reports, Volume *20*

Supplemental Information

APOE4 impacts cortical neurodevelopment and alters network formation in human brain organoids

Karina K. Meyer-Acosta, Eva Diaz-Guerra, Parul Varma, Adyasha Aruk, Sara Mirsadeghi, Aranis Muniz-Perez, Yousef Rafati, Ali Hosseini, Vanesa Nieto-Estevez, Michele Giugliano, Christopher Navara, and Jenny Hsieh

Figure S1 - iPSC characterization

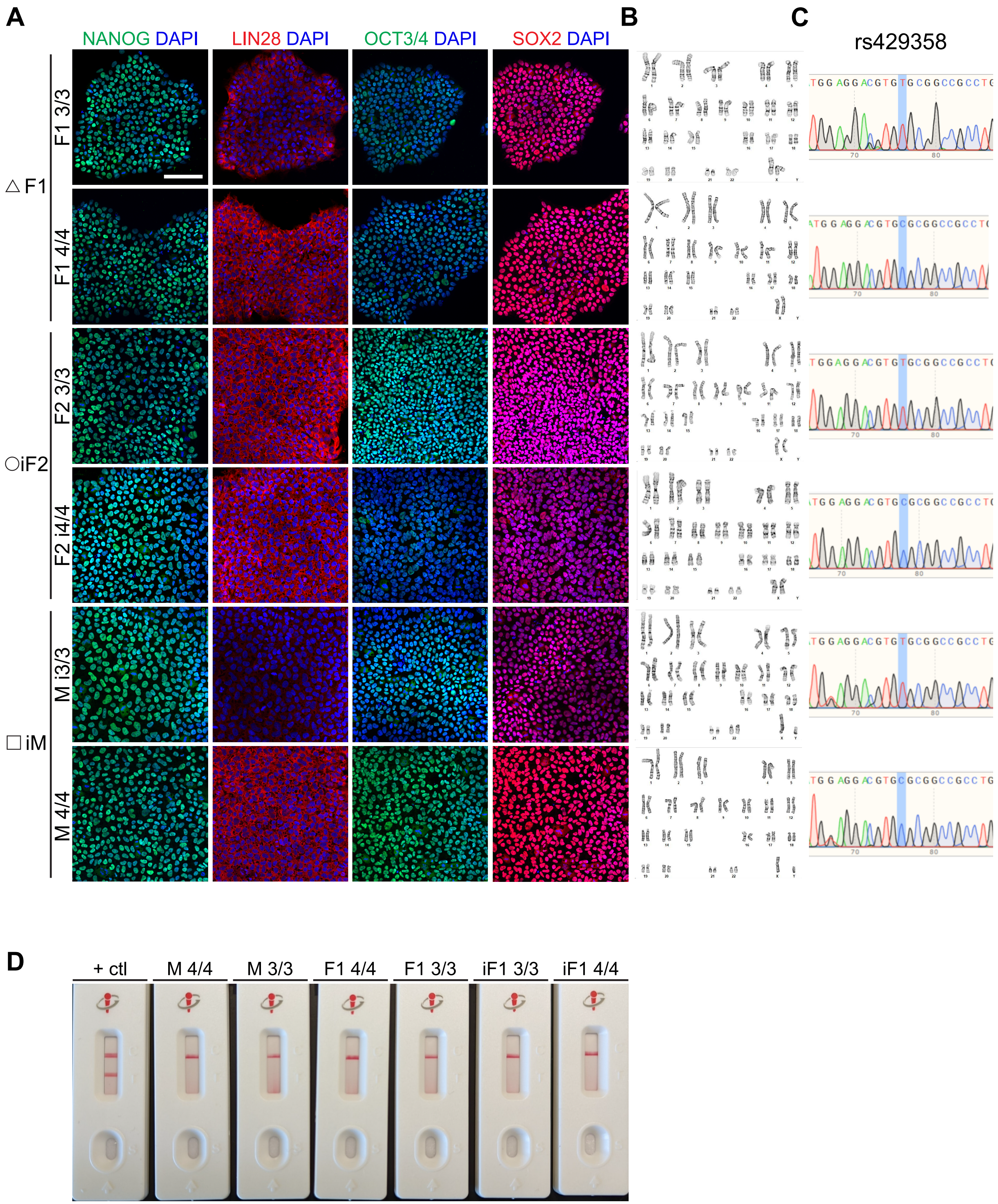

Figure S1. iPSCs characterization.
A) ICC representative images of each iPSC line used in this study showing the expression of the pluripotency markers Nanog, Lin28, OCT3/4, and SOX2. The nucleus was stained with DAPI. Each line was confirmed to have B) a normal karyotype, and C) a correct genotype. D) representative image of routine mycoplasma testing negativity, which are performed prior to and routinely during organoid culture. Scale bar = 100 μm.

Figure S2 - CO 1

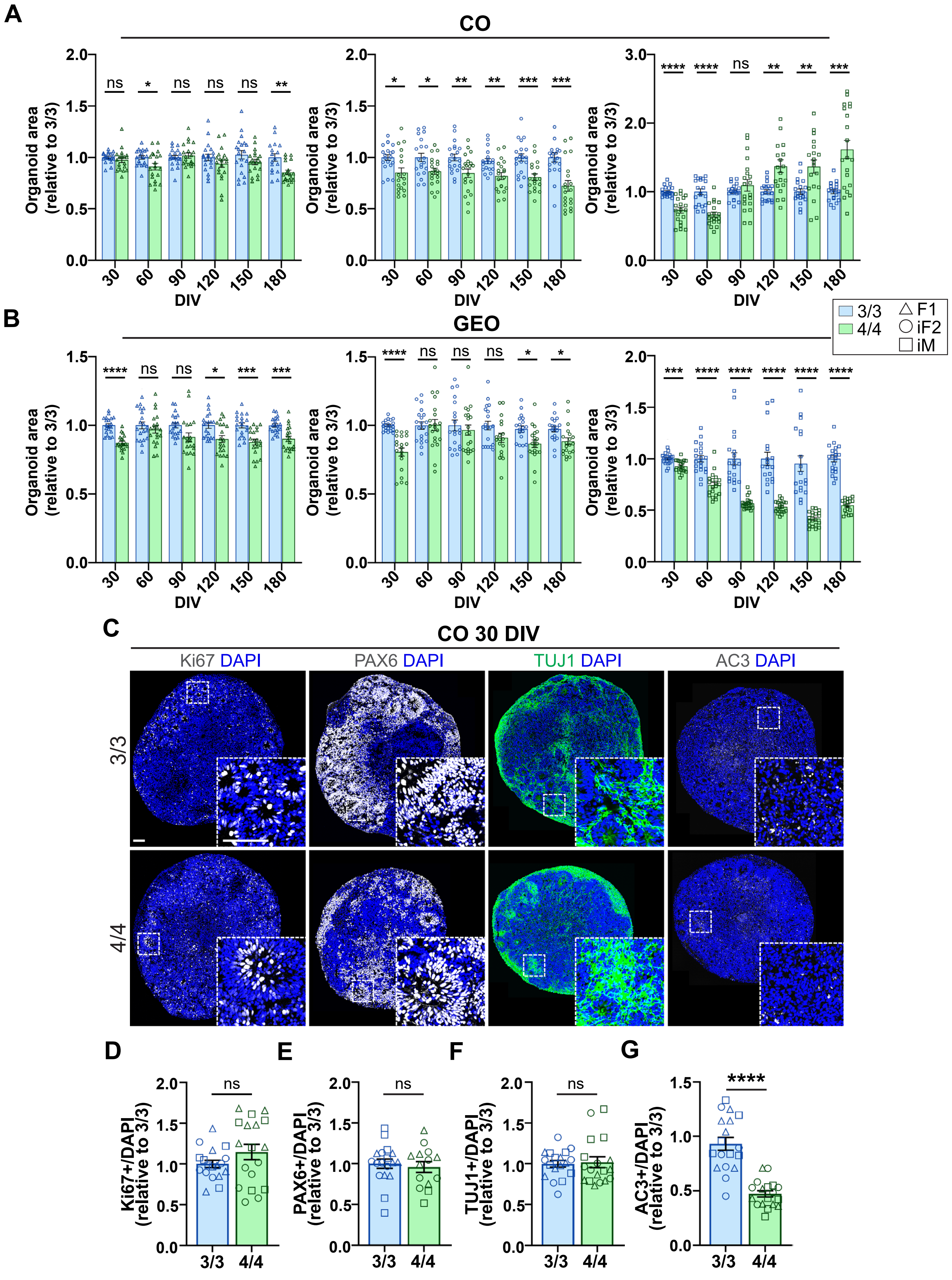

Figure S2. Size and characterization of COs at early time points (Related to Figure 2).
Assessment of organoid size of COs and GEOs across 30-180 DIV and IHC analysis of cellular phenotypes for COs at 30 DIV. (A-B) Organoid area across DIV, represented relative to APOE3/3 for: A) COs and B) GEOs, separated by iPSC line pair. C) Representative IHC images of APOE3/3 and APOE4/4 COs immunostained for Ki67, PAX6, TUJ1, and AC3. (D-G) Quantification of marker-positive cells over DAPI, represented relative to APOE3/3: D) Ki67 E) PAX6 F) TUJ1 and G) AC3. Organoid area: N = 57-60 organoids. IHC: N = 16-18 organoids. Data are presented as mean ± SEM, from 3 *APOE3/3* and 3 *APOE4/4* iPSC lines (2 replicates per iPSC line). Unpaired t-tests with Welch's correction were used to determine significance. Scale bar = 100 μm.

Figure S3 - CO 2

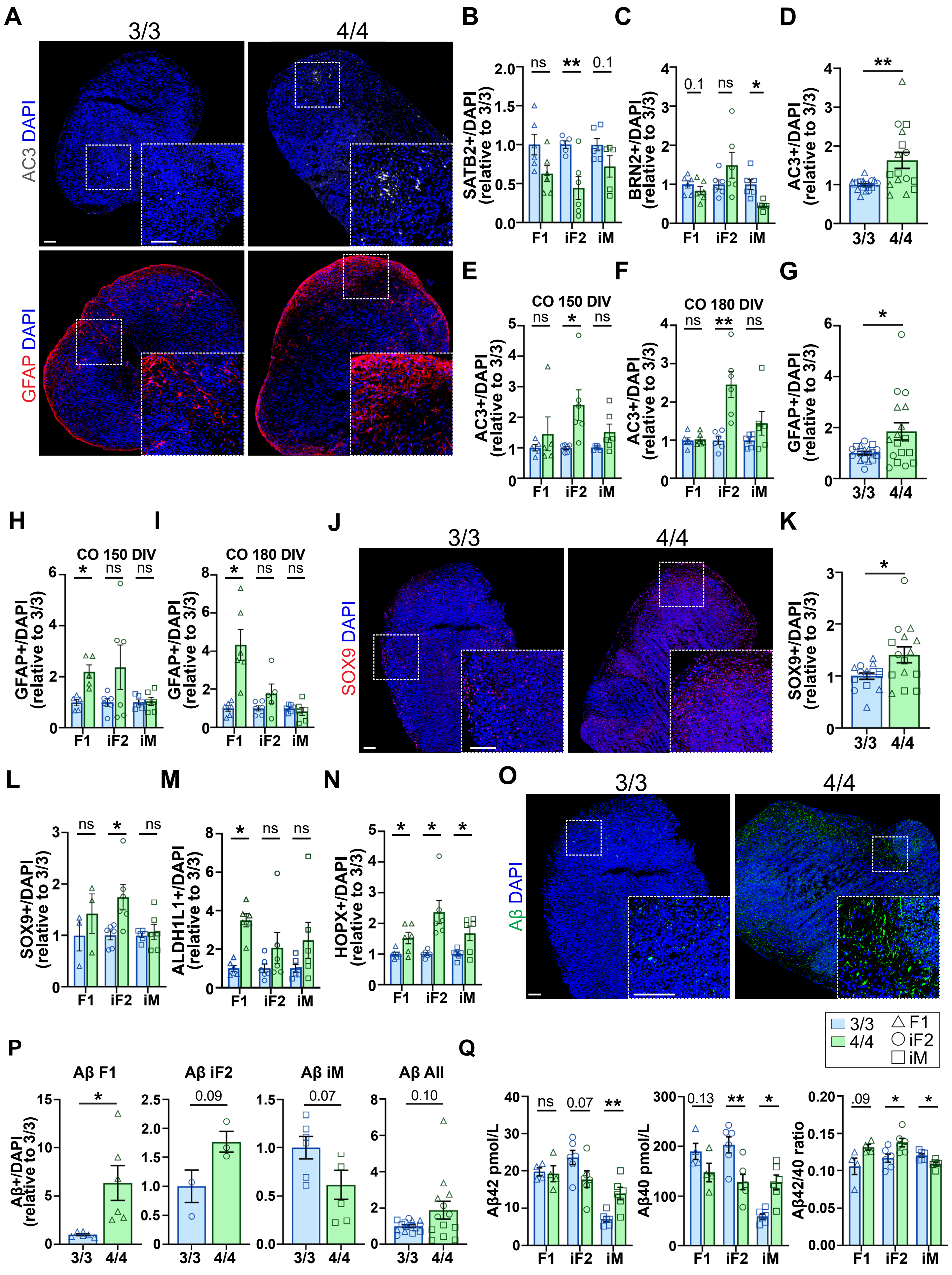

Figure S3. Characterization of COs at gliogenic time points (Related to Figure 2).
Characterization of COs at 150 and 180 DIV to evaluate cell type-specific markers, cell death, and Aβ pathologies via IHC and ELISA. A) Representative IHC images of *APOE3/3* and *APOE4/4* COs immunostained for AC3 and GFAP at 150 DIV. (B-C) Cortical neuron marker analysis corresponding to Figure 2, separated by iPSC line: B) SATB2 and C) BRN2. (D-F) AC3 quantification at 150 and 180 DIV: D) Combined analysis at 150 DIV. E) 150 DIV, separated by line. F) 180 DIV, separated by line. (G-I) GFAP IHC at 150 and 180 DIV: G) Combined analysis at 150 DIV. H) 150 DIV, separated by line. I) 180 DIV, separated by line corresponding to Figure 2. (J-L) SOX9 IHC at 180 DIV: J) Representative images of SOX9 in *APOE3/3* and *APOE4/4* COs. K) Combined analysis. L) Separated by line. (M-N) IHC analysis of other glial markers separated by line corresponding to Figure 2: M) ALDH1L1 and N) HOPX. O) Representative IHC images of Aβ (D54D2 antibody) in COs at 180 DIV. P) Aβ IHC quantification separated by iPSC line and combined. Q) ELISA from media of secreted Aβ42 and Aβ40 levels and Aβ42/Aβ40 ratio separated by line in COs at 180 DIV. Data are represented as mean ± SEM from 3 *APOE3/3* and 3 *APOE4/4* iPSC lines (2 replicates per iPSC line). IHC: N =16-18 organoids, 3 organoids per replicate. ELISA: N = 16 samples (each pooled from 3–4 organoids). Unpaired t-tests with Welch’s correction were used to determine significance. * $p < 0.05$, ** $p < 0.01$, ns: not significant. Scale bar = 100 μm.

Figure S4 - GEO

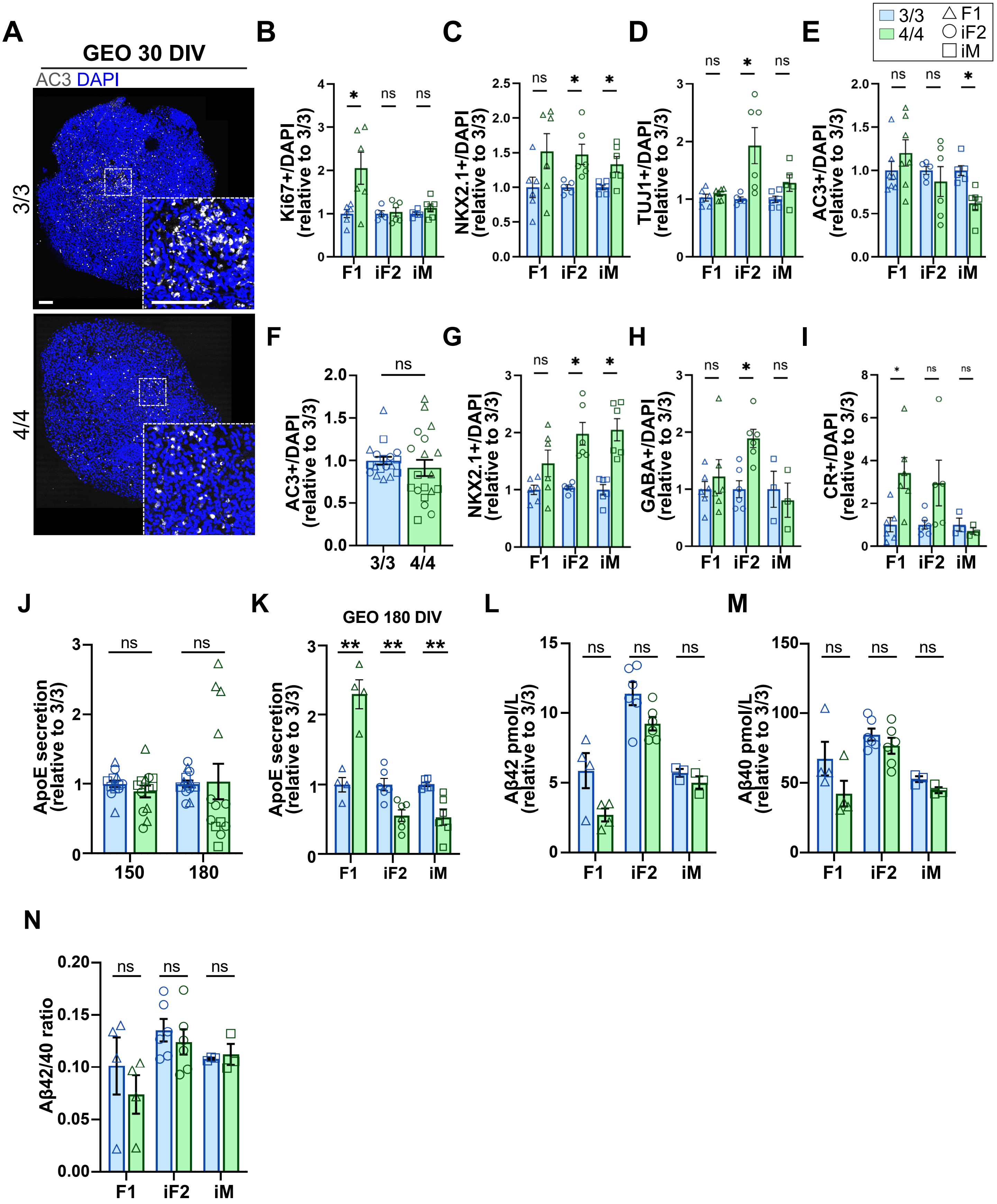

Figure S4. Characterization of GEOs (Related to Figure 3).
Characterization of GEOs for cell death at early (30 DIV) and secreted protein levels at late (150 and 180 DIV) time points. Includes additional IHC graphs corresponding to Figure 3. **A)** Representative IHC images of AC3 in *APOE3/3* and *APOE4/4* GEOs at 30 DIV. **(B-D)** IHC quantification at 30 DIV, separated by iPSC line: **B)** Ki67, **C)** NKX2.1, and **D)** TUJ1 corresponding to Figure 3. **(E-F)** AC3 IHC analysis at 30 DIV: **E)** separated by line and **F)** combined analysis. **(J-K)** ApoE secretion from GEOs measured by ELISA: **J)** Combined analysis at 150 and 180 DIV. **K)** 180 DIV, separated by line. Data shown relative to *APOE3/3*. **(L-N)** Aβ secretion in GEOs at 180 DIV measured by ELISA: **L)** Aβ42, and **M)** Aβ40, and **N)** Aβ42/Aβ40 ratio, separated by line. IHC: N =16-18 organoids (3 *APOE3/3* and 3 *APOE4/4* iPSC lines). ELISA: 1–3 samples per iPSC line, each with 2 replicates. Data are represented relative to APOE3/3 and as mean ± SEM. Unpaired t-tests with Welch's correction were used to determine significance. * $p < 0.05$, ** $p < 0.01$, ns: not significant. Scale bar = 100 μm.

Figure S5 - RNAseq

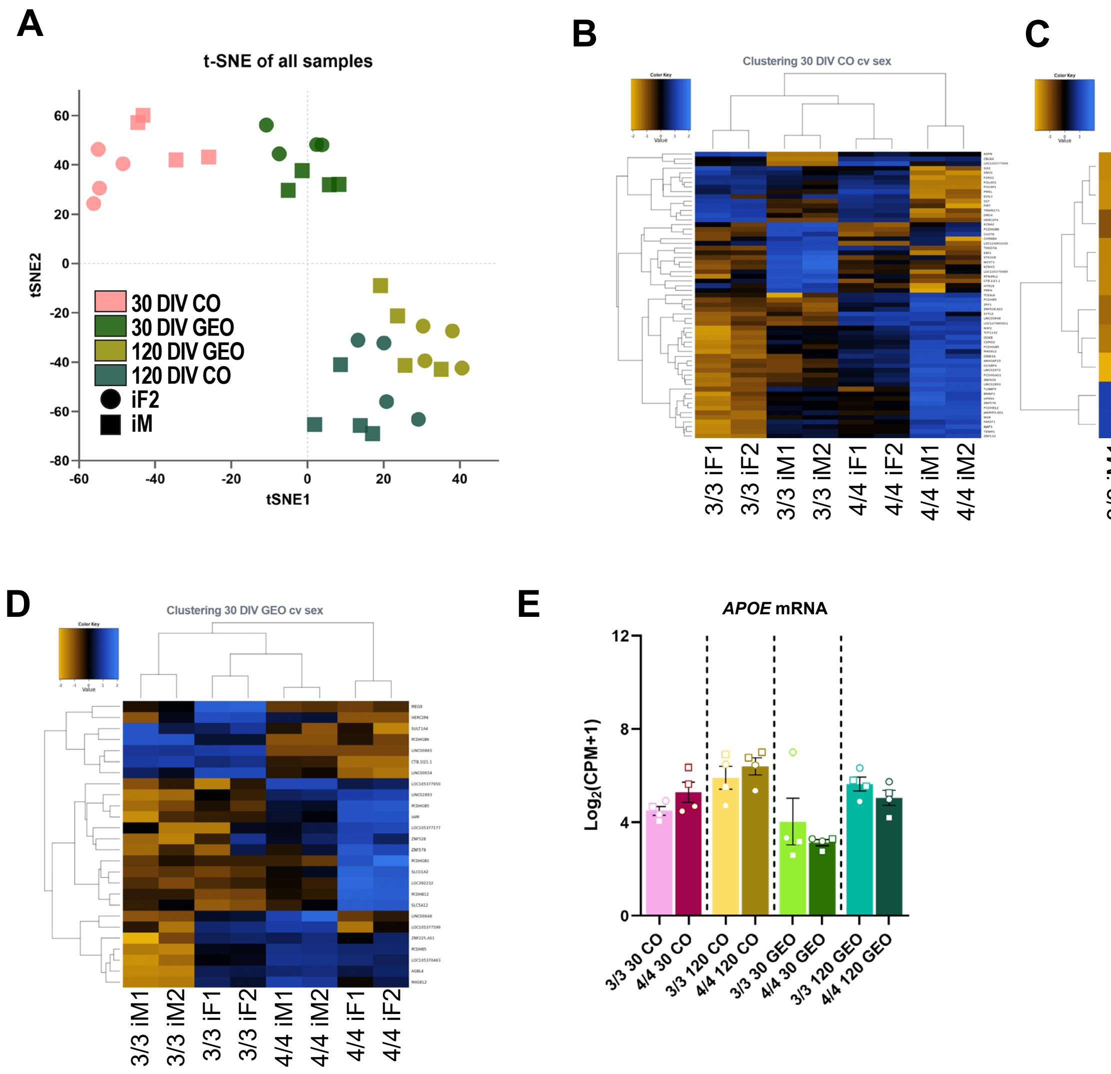

Figure S5. Bulk RNA-seq characterization of COs and GEOs (Related to Figure 4).
Bulk RNA sequencing of COs and GEOs derived from isogenic APOE3/3 and APOE4/4 pairs, collected at 30 and 120 DIV. **A)** t-SNE Scatter plot shows organoid clustering by type and timepoint. Points are colored by organoid type and timepoint. **(B-D)** Heatmap shows clustering analysis of each line and replicate for differentially abundant features in *APOE3/3* vs. *APOE4/4*: **B)** COs at 30 DIV, and **C)** COs at 120 DIV, and **D)** GEOs at 30 DIV. Dendrograms show Euclidean clustering. Data are CPM-normalized, log2-transformed, and z-score scaled. **E)** Barplot shows the $\log_2$(CPM+1) normalized values of *APOE* expression on the y-axis and all conditions on the x-axis. Bars are colored by condition and data is represented as mean ± SEM.

Figure S6 - MEA and gene expression

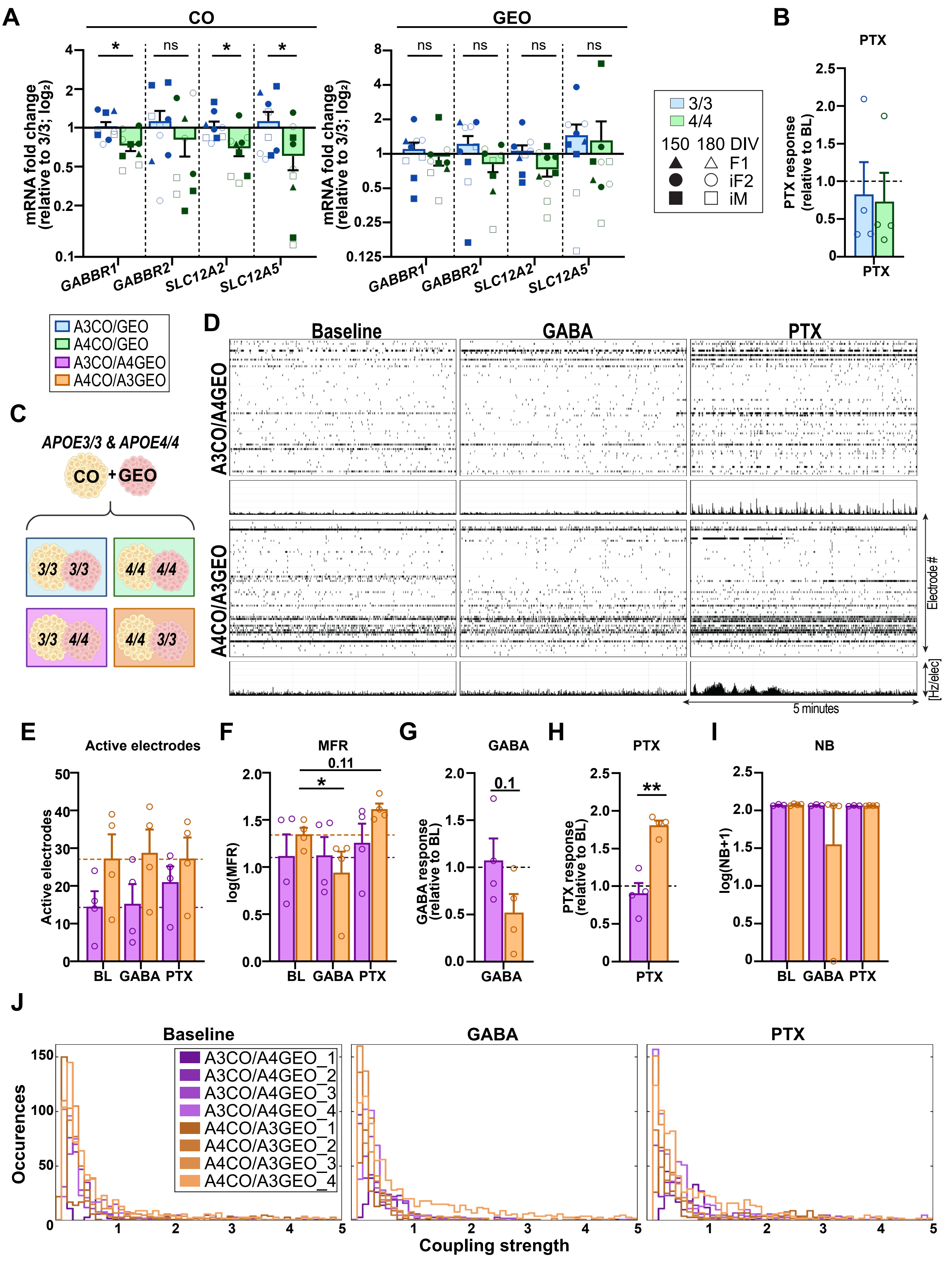

Figure S6. GABA-related gene expression and mixed assembloid MEA analysis (Related to Figure 5).
Assessment of GABA-related gene expression at 150 and 180 DIV in COs and GEOs, and functional activity in mixed-genotype assembloids at 220 DIV. **A)** qRT-PCR analysis of $GABA_B$ receptors (*GABBR1*, *GABBR2*) and Cl^- transporters (*SLC12A2*, *SLC12A5*) in *APOE3/3* and *APOE4/4* COs and GEOs. N = 9 samples (3 *APOE3/3* and 3 *APOE4/4* iPSC lines, 2 replicates per line, 3 organoids per sample). **B)** PTX response for APOE3 and APOE4 matched assembloids, measured as the MFR ratio of PTX over BL for each respective assembloid. **C)** schematic of matched and mix-matched assembloid and corresponding barplot color. **(D-J)** MEA analysis of A3CO and A4CO mixed assembloids. **D)** Raster plots show spikes across 60 electrodes over a representative 5 minute recording during baseline, GABA, and PTX conditions. Mean firing rate (0–10 Hz/sec) is shown beneath each raster. **(E-I)** Bar plots show: **E)** active electrodes, **F)** MFR (log-transformed), **G)** GABA response, **H)** PTX response, **I)** total network bursts (log transformed: log(NB+1)) in each condition. **J)** Histograms of cross-correlation strength between electrode pairs (x-axis: coupling strength, y-axis: frequency). Only significant coupling peaks are shown. N = 4 assembloids per genotype from isogenic *APOE3/3* and *APOE4/4* iPSC lines. Data are represented as mean ± SEM. 2-way ANOVA with Sidak's multiple comparisons was used for bar plots; unpaired t-tests were used for other comparisons. * $p < 0.05$, ns: not significant.

Supplemental Tables

Table S1: List of primary antibodies used in this study				
**Antibody**	Host Species	Company	Cat. Number	Dilution
**Anti-AC3**	Rabbit	Cell Signaling	9661	1:400
**Anti-ALDH1L1**	Rabbit	Abcam	Ab177463	1:200
**Anti-BRN2**	Mouse	EMD Millipore	MABD51	1:50
**Anti-Calretinin**	Mouse	Swant	CR6B	1:1000
**Anti-D54D2 (Aβ)**	Rabbit	Cell Signaling	8243	1:500
**Anti-GABA**	Rabbit	Sigma	A2052	1:1000
**Anti-GFAP**	Chicken	Millipore Sigma	AB5541	1:1000
**Anti-HucHuD**	Mouse	Invitrogen	A-21271	1:500
**Anti-HOPX**	Rabbit	Sigma	HPA055888	1:500
**Anti-Ki67**	Mouse	BD	550609	1:500
**Anti-Ki67**	Rat	Invitrogen	14-5698-82	1:200
**Anti-LIN28**	Rabbit	Cell Signaling	3978	1:1000
**Anti-MAP2ab**	Mouse	Sigma	M1406	1:500
**Anti-NANOG**	Mouse	Thermoscientific	MA1-017	1:500
**Anti-NKX2.1**	Rabbit	Abcam	ab133737	1:500
**Anti-Oct3/4**	Mouse	Santa Cruz	sc-5279	1:1000
**Anti-PAX6**	Rabbit	Biolegend	901301	1:300
**Anti-SATB2**	Rabbit	Abcam	AB34735	1:500
**Anti-SOX2**	Rabbit	Millipore	AB5603	1:1000
**Anti-SOX9**	Goat	R&D Systems	AF3075	1:500
**Anti-TUJ1**	Mouse	Sigma	T8660	1:400

Table S2: List of primers for RT-qPCR, related to Figure S6		
**Gene**	Forward	Reverse
**GABBR1**	TTCAACTACAACAACCAGACCATTACCG	GCGTCCATGCCATCCGAGAG
**GABBR2**	CCCCTGCGAAGGACAGTGGAG	AACAACCGAACAACATGAGAAGTCCC
**GAPDH**	GGAAGCTTGTCATCAATGGAAATC	TCAGCAGAGGGGGCAGAGAT
**SLC12A2**	AACGCTGTTGCAGTTGCTATGTATGTG	AGATACCTAAAAGAATCACGACTGTAATGGC
**SLC12A5**	CTGCAGAACATCTTTGGCGTCATC	CAGCAGGCACAACACCATTCGT

Table S3: Software and statistics, related to Figures 4 and 5		
	Reference	URL
**nf-core-rnaseq (pipeline v3.12.0)**	(Ewels et al., 2020; Harshil Patel, 2024)	https://nf-co.re/rnaseq
**Trim Galore**	(FelixKrueger/TrimGalore)	https://www.bioinformatics.babraham.ac.uk/projects/trim_galore/
**STAR**	(Dobin et al., 2012)	10.1093/bioinformatics/bts635
**RSEM**	(Li and Dewey, 2011)	10.1186/1471-2105-12-323
**DESeq2**	(Love et al., 2014)	10.18129/B9.bioc.DESeq2
**fGSEA**	(Korotkevich et al., 2021)	http://bioconductor.org/packages/fgsea/
**MSigDB**	(Dolgalev, 2021; Liberzon et al., 2015; Subramanian et al., 2005)	https://www.gsea-msigdb.org/gsea/msigdb/human/genesets.jsp?collection=C8
**msigdb R**	(Dolgalev, 2021)	https://CRAN.R-project.org/package=msigdbr
**edgeR**	(Robinson et al., 2009)	10.18129/B9.bioc.edgeR
**Rtsne**	(Krijthe, 2015)	https://github.com/jkrijthe/Rtsne
**Pluto**	pluto.bio	https://pluto.bio
**ImageJ**	(Schneider et al., 2012)	https://imagej.nih.gov/ij/
**Matlab**	The MathWorks Inc., Natick, Massachusetts	https://www.mathworks.com
**Julia**	(Bezanson et al., 2017)	https://julialang.org/
**QSpikeTools**	(Mahmud et al., 2014)	https://github.com/mgiugliano/SpiQ

Supplemental methods

iPSC characterization continued:

APOE4/4 female iPSC line (CW50129) was sourced from CIRM. An iPSC line was generated by the UTSA stem cell core from fibroblast samples (Coriell) using Cytotune-iPS Sendai Reprogramming kit (Thermofisher). We also confirmed no integration of reprogramming genes. All iPSCs were expanded to create stocks from 3-4 passages then karyotyped (Wicell), and experiments were performed within 5 passages of karyotype. For pluripotency, iPSC's were grown on coverslips in a 24-well plate and were fixed at 75% confluence for immunocytochemistry (see below). All iPSC lines were negative for mycoplasma throughout the study using either genomic PCR (Cat No. MP0035-1KT, Millipore sigma) or supernatant (Cat No. rep-mys-20, Invitrogen). iPSCs were maintained without antibiotics with no visible signs of microbial presence.

Detailed organoid generation protocol:

iPSCs were seeded in a 6-well plate at 275,000 cells per well. 12-24 hours prior to organoid generation, 1% DMSO was added during routine feeding to enhance the differentiation of iPSCs (Chetty et al., 2013). On day 0 of differentiation, iPSCs at 70% confluence were detached and dissociated with Accutase (Sigma-Aldrich) and washed with mTESR. iPSCs were seeded at 9,000 cells per well of an ultra-low attachment 96-well round bottom plate (Corning Cat. No. 7007) in mTeSR containing Y27632 (20 µM), and medium changes were performed daily. On Day 1, media was changed to neural induction medium TeSR™-E6 medium (Cat. No. 05946, Stemcell Technologies). On days 1-5, Dual-SMAD inhibition was performed using SMAD inhibitors dorsomorphin (2.5 µM, Sigma) and SB-431542 (10 µM, Tocris), and wnt-inhibitor XAV 939 (1.2 µM, Tocris) was added to enhance forebrain differentiation. On days 6-24, media was replaced with neural medium (Neurobasal A, B-27 without Vitamin A, Glutamax, penicillin/streptomycin) containing the growth factors, bFGF (20 ng/ml, Peprotech) and EGF (20 ng/ml, Peprotech). On day 15, organoids were transferred to a 24-well plate (Corning Cat. No. 3473) and were maintained on an orbital shaker (80 RPM) to promote oxygenation. On day 25, media was replaced with neural medium containing BDNF (20 ng/ml, Peprotech) and NT-3 (20 ng/ml, Peprotech) until day 42. For ganglionic eminence organoid generation, Wnt inhibitor IWP-2 (5 µM, Selleckchem) was added on days 4-22, and the SMO pathway activator, SAG (100 nM, Selleckchem) on days 12-22.

IHC and sample preparation

For iPSCs: iPSCs were plated on glass coverslips in a 24-well plate. When cells reached 60-70% confluence, they were fixed with 4% paraformaldehyde (PFA) for 15 minutes and washed 3 times with PBS. Coverslips were stained using the same protocol as described below for organoids. **For organoids:** At each time point, organoids were harvested and fixed in 4% PFA for 1-2 hours at room temperature or overnight at 4 °C, then incubated at 4 °C in 30% sucrose until they sank (24-48 hours). Organoids are embedded in OCT compound and frozen on dry ice. Organoids were cryosectioned at 14-µm, in serial, on glass slides. For IHC, organoids were incubated in blocking buffer (1X Carbo-Free Blocking solution (Vector labs, Cat. No. SP-5040-125), 0.3% Triton X-100 in TBS) for 1 hour at room temperature (RT), and primary antibodies (See Table S1) overnight at 4 °C in a humidified chamber. After 3 TBS washes, slides were incubated with secondary antibodies (Jackson Immunoresearch, 1:400, or Alexafluor 488, 1:1000, or Alexafluor 647, 1:400) for 2 hours at RT. Slides were washed, 4',6-diamidino-2-phenylindole (DAPI; Sigma, Cat. No. D9542) was added to the second wash to label nuclei, then coverslipped using polyvinyl Alcohol solution (PVA; Sigma, Cat. No. BP168-122). **Confocal image processing for presentation:** To improve image presentation in figures, the brightness and contrast was adjusted using ImageJ fiji software. Adjustments were made equally in APOE3/3 and APOE4/4 for each marker.

Bulk-RNA sequencing details

75 paired-end RNAseq (2x75bp flowcell) was performed by the UTSA genomics core on all 32 samples at the same time with Element Biosciences AVITI DNA Sequencer, using the Freestyle Cloudbreak chemistry at a depth of 25-30 million reads per sample. Analysis performed and plots created using Pluto (https://pluto.bio). paired_end FASTQ files were processed using the nf-core-rnaseq pipeline (v3.12.0)(auto strandedness)(Ewels *et al.*, 2020). Adapter sequences were removed with Trim Galore. Reads were aligned with STAR (Dobin *et al.*, 2012) to GRCh38 (NCBI, p.14, release 110) and quantified to gene counts using RSEM (Li and Dewey, 2011). **Differential gene expression analysis:** Differential expression analysis was performed with the DESeq2 R package (Love *et al.*, 2014), which tests for differential expression based on a model using the negative binomial distribution. **Gene set enrichment analysis (GSEA):** was performed using the fgsea R package and the fgsea Multilevel() function (Korotkevich *et al.*, 2021). The $\log_2$ fold change from APOE3/3 and APOE4/4 differential expression comparisons were used to rank genes. C5: Gene Ontology gene sets - biological process gene set collection from the Molecular Signatures Database (MSigDB) (Dolgalev, 2021) was curated using the msigdbr R package(Liberzon *et al.*, 2015; Subramanian *et al.*, 2005). Prior to running GSEA, the list of gene sets was

filtered to include only gene sets with between 5 and 1000 genes. **Clustering analysis:** Clustering analysis was performed for each cell line and replicate using differentially abundant features in the comparison of *3/3* vs. *4/4* for each time point and organoid type. Features were filtered using an adjusted p-value ≤ 0.01 and log_2 fold change threshold of 1 (showing features that are both positive and negative). Prior to plotting, the data was counts per million (CPM)-normalized, log_2-transformed, and z score-transformed. **Venn diagram:** Overlap genes analysis was performed using the following gene sets: CO 30 DIV and GEO 30 DIV. For gene set CO 30 DIV, differential genes were defined as genes with an adjusted p-value of less than or equal to 0.05, and a fold change greater than 1.2. For gene set GEO 30 DIV, differential genes were defined as genes with an adjusted p-value of less than or equal to 0.05, and a fold change greater than 1.2. **APOE CPM bar plot:** Bar plot showing the counts per million (CPM)-normalized values for APOE on the y-axis and *3/3* and *4/4* for each time point and organoid type on the x-axis. CPM values were calculated using the cpm() function in the edgeR R package (Robinson *et al.*, 2009) with log=F. Bars are drawn at the average value for each Group and are colored according to Group. Error bars represent the average +/- standard error of the mean (SEM). Values on the y-axis are displayed on a symmetrical log_{10} scale. **t-distributed stochastic neighbor embedding analysis (t-SNE):** Barnes-Hut t-Distributed Stochastic Neighbor Embedding (t-SNE)(Laurens van der Maaten; 2014) was performed by applying the Rtsne() R function (Krijthe, 2015) to raw values for all 43,236 targets in the experiment and all samples. An initial principal components analysis (PCA) step was run prior to t-SNE. The perplexity parameter used was 10.3.

Detailed MEA recording and analysis:

All recordings were carried out inside a humid (i.e., 95% R.H.) incubator at 37℃ and 5% CO2. Organoids were maintained using BrainPhys™ hPSC Neuron kit (05795; STEMCELL Technology) for a week prior to recording session. For each session, individual organoids were placed on 3D – MEA (60-3DMEA200/12/80iR-Ti, Multichannel system, Harvard Bioscience). To ensure optimal attachment to the MEA surface, organoids were immersed in 200 µL of BrainPhys™. Sequential recordings were performed as follows: baseline, 20 µM GABA (56-12-2, Sigma-Aldrich), and 58 µM picrotoxin (124-87-8, Sigma-Aldrich), for 30 minutes each condition, with recording started 10 minutes after drug application. Fluorinated Teflon thin film (MEA-MEM-set5, Multichannel systems, Harvard Bioscience) was used to seal the chip, to block evaporation and contamination. Raw electrical potentials were amplified using electronic amplifier (ME2100-Mini, Multichannel systems, Harvard Bioscience), sampled at 25 kHz/channel, and digitized at 16-bit resolution. Data were stored on disk via software (Experimenter, Multichannel systems, Harvard Bioscience). To detect the time of occurrence of putative action potentials (i.e. spike times), a peak detection algorithm with adaptive threshold was employed, for spontaneous network-wide synchronization of spike times (i.e. network bursts) could be detected and quantified (Mahmud *et al.*, 2014; Quiroga et al., 2004). The events were visualized as a raster plot and further analyzed by spike train analysis (Mahmud *et al.*, 2014; Quiroga *et al.*, 2004). Interaction between electrode pairs was derived using cross-correlation analysis of spike times (Knox, 1981), limited to inter-spike delays less than 500 ms and quantified by 3 ms bins. Each cross correlogram was divided by the square root of spike number in a spike train to account for firing rate modulation. The peak value from the cross-correlogram represented connectivity strength of the electrode pairs. Peak value distribution across experimental conditions were generated to facilitate a comparison of coupling strength. To assess the significance of these peaks (coupling strength), their corresponding inter-spike intervals (ISI) were randomly shuffled to achieve surrogate spike times with identical distribution of ISI. The peaks were considered significant if they showed values larger than the mean plus 3 standard deviations of the cross-correlogram of the surrogate.

Additional Statistics

To determine whether *APOE4/4* lines were significantly different from one another for each IHC marker, statistics were performed between all markers separated by line using a Brown-Forsythe and Welch's one-way ANOVA. If either test was significant, we calculated the p-value for each *APOE4/4* comparison using Dunnett's T3 correction for multiple comparisons.

Supplemental References

(FelixKrueger/TrimGalore).

Bezanson, J., Edelman, A., Karpinski, S., and Shah, V.B. (2017). Julia: A Fresh Approach to Numerical Computing. SIAM Review 59, 65-98. 10.1137/141000671.

Chetty, S., Pagliuca, F.W., Honore, C., Kweudjeu, A., Rezania, A., and Melton, D.A. (2013). A simple tool to improve pluripotent stem cell differentiation. Nat Methods 10, 553-556. 10.1038/nmeth.2442.

Dobin, A., Davis, C.A., Schlesinger, F., Drenkow, J., Zaleski, C., Jha, S., Batut, P., Chaisson, M., and Gingeras, T.R. (2012). STAR: ultrafast universal RNA-seq aligner. Bioinformatics 29, 15-21. 10.1093/bioinformatics/bts635.

Dolgalev, I. (2021). msigdbr: MSigDB Gene Sets for Multiple Organisms in a Tidy Data Format. .

Ewels, P.A., Peltzer, A., Fillinger, S., Patel, H., Alneberg, J., Wilm, A., Garcia, M.U., Di Tommaso, P., and Nahnsen, S. (2020). The nf-core framework for community-curated bioinformatics pipelines. Nature Biotechnology 38, 276-278. 10.1038/s41587-020-0439-x.

Harshil Patel, P.E., Jonathan Manning, Maxime U Garcia, Alexander Peltzer, Rickard Hammarén, Olga Botvinnik, Adam Talbot, Gregor Sturm, nf-core bot, Matthias Zepper, Denis Moreno, Pranathi Vemuri, Mahesh Binzer-Panchal, Ezra Greenberg, silviamorins, Lorena Pantano, Robert Syme, Gavin Kelly, Friederike Hanssen, James A. Fellows Yates, Jose Espinosa-Carrasco, rfenouil, Luke Zappia, Chris Cheshire, Edmund Miller, marchoeppner, Peng Zhou, Sarah Guinchard, Gisela Gabernet (2024). nf-core/rnaseq (latest version).

Knox, C.K. (1981). Detection of neuronal interactions using correlation analysis. Trends in Neurosciences 4, 222-225. https://doi.org/10.1016/0166-2236(81)90070-9.

Korotkevich, G., Sukhov, V., Budin, N., Shpak, B., Artyomov, M.N., and Sergushichev, A. (2021). Fast gene set enrichment analysis. bioRxiv, 060012. 10.1101/060012.

Krijthe, J.H. (2015). Rtsne: T-Distributed Stochastic Neighbor Embedding using Barnes-Hut Implementation.

Laurens van der Maaten, G.H. Visualizing Data using t-SNE. Journal of Machine Learning Research 9, 2579-2605, 86.

Laurens van der Maaten, G.H. (2014). Accelerating t-SNE using Tree-Based Algorithms. Journal of Machine Learning Research 15, 3221-3245, 93.

Li, B., and Dewey, C.N. (2011). RSEM: accurate transcript quantification from RNA-Seq data with or without a reference genome. BMC Bioinformatics 12, 323. 10.1186/1471-2105-12-323.

Liberzon, A., Birger, C., Thorvaldsdóttir, H., Ghandi, M., Mesirov, J.P., and Tamayo, P. (2015). The Molecular Signatures Database (MSigDB) hallmark gene set collection. Cell Syst 1, 417-425. 10.1016/j.cels.2015.12.004.

Love, M.I., Huber, W., and Anders, S. (2014). Moderated estimation of fold change and dispersion for RNA-seq data with DESeq2. Genome Biology 15, 550. 10.1186/s13059-014-0550-8.

Mahmud, M., Pulizzi, R., Vasilaki, E., and Giugliano, M. (2014). QSpike tools: a generic framework for parallel batch preprocessing of extracellular neuronal signals recorded by substrate microelectrode arrays. Frontiers in Neuroinformatics 8. 10.3389/fninf.2014.00026.

Quiroga, R.Q., Nadasdy, Z., and Ben-Shaul, Y. (2004). Unsupervised spike detection and sorting with wavelets and superparamagnetic clustering. Neural Comput 16, 1661-1687. 10.1162/089976604774201631.

Robinson, M.D., McCarthy, D.J., and Smyth, G.K. (2009). edgeR: a Bioconductor package for differential expression analysis of digital gene expression data. Bioinformatics 26, 139-140. 10.1093/bioinformatics/btp616.

Schneider, C.A., Rasband, W.S., and Eliceiri, K.W. (2012). NIH Image to ImageJ: 25 years of image analysis. Nature Methods 9, 671-675. 10.1038/nmeth.2089.

Subramanian, A., Tamayo, P., Mootha, V.K., Mukherjee, S., Ebert, B.L., Gillette, M.A., Paulovich, A., Pomeroy, S.L., Golub, T.R., Lander, E.S., and Mesirov, J.P. (2005). Gene set enrichment analysis: A knowledge-based approach for interpreting genome-wide expression profiles. Proceedings of the National Academy of Sciences 102, 15545-15550. doi:10.1073/pnas.0506580102.
