# Supplementary material for: APOE4 impacts cortical neurodevelopment and alters network formation in human brain organoids
Source: Stem Cell Reports. 2025 Jun 19;20(7):102537. doi: 10.1016/j.stemcr.2025.102537 (PMC12277819; doi:10.1016/j.stemcr.2025.102537)
Supplement: Document S2. Article plus supplemental information [file mmc4.pdf]

Stem Cell Reports
Article

OPEN ACCESS

APOE4 impacts cortical neurodevelopment and alters network formation in human brain organoids

Karina K. Meyer-Acosta,[1,2] Eva Diaz-Guerra,[1,2] Parul Varma,[1,2] Adyasha Aruk,[1,2] Sara Mirsadeghi,[1,2] Aranis Muniz-Perez,[1,2] Yousef Rafati,[1,2] Ali Hosseini,[4] Vanesa Nieto-Estevez,[1,2] Michele Giugliano,[5] Christopher Navara,[1,2,3] and Jenny Hsieh[1,2,6,*]

[1]Department of Neuroscience, Developmental and Regenerative Biology, The University of Texas at San Antonio, San Antonio, TX, USA
[2]Brain Health Consortium, The University of Texas at San Antonio, San Antonio, TX, USA
[3]Stem Cell Core, The University of Texas at San Antonio, San Antonio, TX, USA
[4]International School of Advanced Studies, Neuroscience Area, V. Bonomea 265, 34136 Trieste, Italy
[5]Department of Biomedical, Metabolic & Neural Sciences, University of Modena and Reggio Emilia, V. Campi 287, 41125 Modena, Italy
[6]Lead contact
*Correspondence: jenny.hsieh@utsa.edu
https://doi.org/10.1016/j.stemcr.2025.102537

SUMMARY

Apolipoprotein E4 (*APOE4*) is the leading genetic risk factor for Alzheimer's disease. While most studies examine the role of *APOE4* in aging, *APOE4* causes persistent changes in brain structure as early as infancy and is associated with altered functional connectivity that extends beyond adolescence. Here, we used human induced pluripotent stem cell-derived cortical and ganglionic eminence organoids (COs and GEOs) to examine *APOE4*'s influence during the development of cortical excitatory and inhibitory neurons. We show that *APOE4* reduces cortical neurons and increases glia by promoting gliogenic transcriptional programs. In contrast, *APOE4* increases proliferation and differentiation of GABAergic progenitors resulting in early and persistent increases in GABAergic neurons. Multi-electrode array recordings in assembloids revealed that *APOE4* disrupts neural network function resulting in heightened excitability and synchronicity. Together, our data provide new insights on how *APOE4* influences cortical neurodevelopmental processes and the establishment of functional networks.

INTRODUCTION

Alzheimer's disease (AD) is the most common form of dementia, characterized by progressive cognitive decline, with sporadic AD accounting for 95% of all cases (Harman, 2006). AD pathology begins decades before cognitive symptoms, yet early phenotypic changes preceding AD pathology are unknown (Holtzman et al., 2011). Apolipoprotein E4 (*APOE4*) is the leading genetic risk factor for AD, increasing risk 3- to 12-fold (Karch et al., 2014). While *APOE4* has been extensively studied in the context of AD and aging, its genetic influence on brain structure and function related to AD susceptibility has received limited attention. Thus, defining the influence of *APOE4* on brain development and function may provide insight into *APOE4*-mediated AD susceptibility.

Human *APOE4* carriers exhibit altered brain structure and function as early as infancy, with persistent regional effects across development. In infants and toddlers, gray matter volume (GMV) and myelin water fractionation are decreased in the precuneus, temporal, and occipitotemporal regions, areas vulnerable to AD, and increased in the parietal, occipital, and frontal regions in *APOE4* carriers (Dean et al., 2014; Knickmeyer et al., 2014). *APOE4* infants excel in early cognition but later show lower intelligence quotient, attention, and memory, suggesting that early structural changes influence cognition (Chang et al., 2016; Remer et al., 2020; Reynolds et al., 2019). Regional effects of *APOE4* in infants persist into childhood and young adulthood, with increase volume and thickness in parietal, occipital, and frontal regions and reductions in the precuneus and temporal/occipitotemporal regions (Chang et al., 2016; Knickmeyer et al., 2014; Remer et al., 2020; Shaw et al., 2007). Thinning of entorhinal cortex and orbitofrontal cortex, AD-associated regions, emerges in childhood and persists in adulthood (Chang et al., 2016; O'Dwyer et al., 2012; Shaw et al., 2007). Functionally, studies show that *APOE4* increases cortical and hippocampal co-activation and functional connectivity during memory encoding and at rest (Cacciaglia et al., 2020; Filippini et al., 2009; O'Dwyer et al., 2012; Zheng et al., 2018). These studies suggest that early alterations in brain structure with *APOE4* may lead to compensatory mechanisms, which may contribute to later AD vulnerability.

Within the central nervous system, ApoE is primarily expressed in glial cells and is highest in astrocytes, adult and embryonic neural stem cells (NSCs), and neural progenitors (NPs) (Kim et al., 2009; Yuzwa et al., 2017). *Apoe* deletion disrupts NSC maintenance, leading to early postnatal neuronal expansion, NP depletion, and a shift toward gliogenesis at the expense of neurogenesis in adult mice (Yang et al., 2011). Similarly, adult mice expressing humanized *APOE4* exhibit a shift toward gliogenesis at the expense of neurogenesis, which correlates with cognitive deficits

© 2025 The Author(s).
Published by Elsevier Inc. on behalf of International Society for Stem Cell Research.
This is an open access article under the CC BY license (http://creativecommons.org/licenses/by/4.0/).

and reduced responsiveness to GABA inputs, ultimately leading to neuronal hyperexcitability and synchronicity (Area-Gomez et al., 2020; Li et al., 2009). Notably, pharmacological enhancement of GABAergic signaling or interneuron transplantation restores adult neurogenesis and cognitive function (Gillespie et al., 2016; Knoferle et al., 2014; Li et al., 2009; Tong et al., 2016). Collectively, these studies identify a crucial role for ApoE in embryonic and adult neurogenesis.

There are species-specific differences between mouse and human brains, such as increased neuron diversity and ApoE modulation (Maloney et al., 2007; Rakic, 2009). In the human neocortex, outer radial glia (oRG), a population of embryonic NSCs absent in mice, facilitate a second wave of neurogenesis and give rise to both neurons and glia (Hansen et al., 2010). Single-cell RNA sequencing shows that embryonic NSCs and oRG resemble adult NSCs, suggesting that *APOE4*'s influence on adult neurogenesis may extend to embryonic neurogenesis (Baig et al., 2024). To our knowledge, the effect of *APOE4* on embryonic neurogenesis has not been explored in a human-relevant model.

Human induced pluripotent stem cell (iPSC) models have been employed to investigate *APOE4*'s effect on a variety of cell types in the context of AD (Lin et al., 2018; Meyer et al., 2019; Wang et al., 2018; Zhao et al., 2020). One study found that *APOE4* decreased GABAergic neurons, but not glutamatergic neurons, which is consistent with mouse studies (Wang et al., 2018). Additionally, *APOE4* is associated with accelerated differentiation and maturation of mixed NPs, leading to increased functional neuron maturation (Lin et al., 2018; Meyer et al., 2019). *APOE4*'s influence on embryonic neurodevelopment remains unexplored.

We hypothesized that *APOE4* alters neurogenesis and gliogenesis in a neural subtype-specific manner, affecting the maturation and composition of neurons and glia during development. We then reasoned that developmental changes may impact neuronal excitability and network formation. To test this, we used human iPSCs to generate regionalized neural organoids patterned toward the cortex (COs) and ganglionic eminence (GEOs), enriched in excitatory and inhibitory neurons, respectively (Birey et al., 2017). We used immunohistochemistry (IHC) at time points aligning with human neurogenic and gliogenic stages. To assess *APOE4*'s functional influence on network formation, we performed three-dimensional (3D) multi-electrode array (MEA) recordings in fused CO-GEOs (assembloids) at later stages.

Our findings reveal that *APOE4* decreases cortical excitatory neurons while increasing astrocytes and oRG at gliogenic stages, coinciding with elevated cell death in COs. Gene expression analysis showed enrichment for neurodevelopmental and gliogenic transcriptional profiles in *APOE4* COs at neurogenic and gliogenic stages. In GEOs, *APOE4* accelerated neural differentiation of GABAergic NPs during neurogenic stages, increasing mature neurons at later stages. *APOE4* COs and GEOs exhibited early enrichment for receptor-related gene sets, suggesting early neuronal maturation. Lastly, *APOE4* dysregulated GABA-related genes in COs, associated with altered GABA function and heightened synchronicity in assembloids. In summary, *APOE4* differentially influences neural subtypes, disrupting GABA signaling and network patterns in a manner reminiscent of hyperexcitability. These findings support our hypothesis that *APOE4* modulates cortical neurodevelopment, with functional consequences on network formation.

RESULTS

APOE4 promotes gliogenesis while decreasing excitatory neuron subpopulations

To study whether *APOE4* differentially affects the development of neural subtypes, we generated COs and GEOs using a modified protocol adapted from the Pasca lab (Birey et al., 2017). Neural differentiation was induced through dual SMAD inhibition to generate COs and, with the addition of ventralizing factors, GEOs from iPSCs (Figure 1A). Given the heterogeneity imposed by genetic background, we used 1 female and 1 male isogenic pair of human iPSCs in addition to 1 control *APOE3/3* (3/3) and 1 patient with AD *APOE4/4* (4/4) female lines, totaling 6 iPSC lines with three lines per genotype (Table 1). All iPSC lines expressed pluripotency markers (Figure S1A), had a normal karyotype (Figure S1B), and were confirmed for *APOE* genotype (Figure S1C). Isogenic lines did not harbor any off-target mutations (Nimsanor et al., 2016; Peitz et al., 2018). All iPSC lines were confirmed to be mycoplasma-negative before organoid generation and were checked routinely (Figure S1D).

To determine whether *APOE4* altered growth during organoid development, COs and GEOs were imaged every 30 days until 180 days *in vitro* (DIV) using bright-field microscopy (Figure 1B). In COs, we observed an overall size reduction at 30 and 60 DIV with *APOE4* (Figure 1C); however, there was no *APOE4*-mediated size effect at later time points due to significant line differences (Figure S2A). In GEOs however, *APOE4* reduced size at all time points analyzed, in all lines (Figures 1D and S2B). This suggests that *APOE4* mediates region-specific alterations in growth, possibly related to cellular composition, proliferation, differentiation, and/or survival.

We then used IHC to probe COs for proliferation, differentiation, and cell death at the earliest time point.

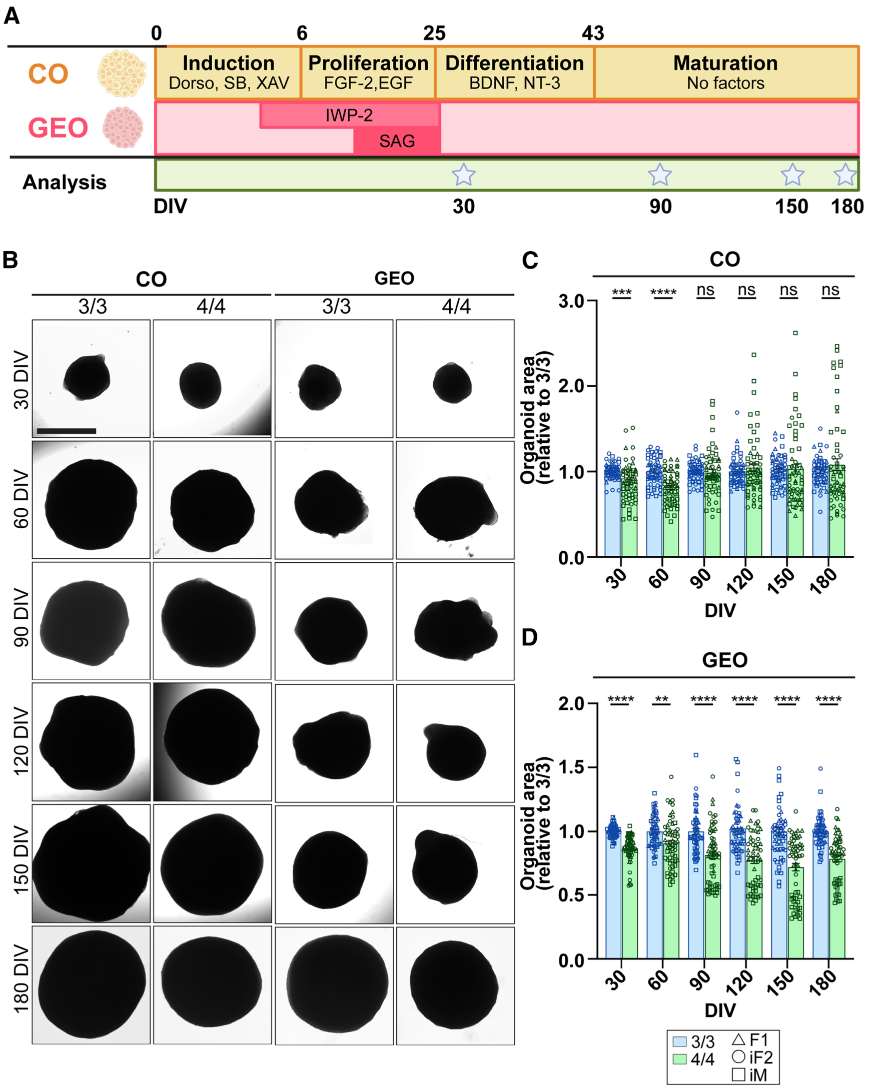

Figure 1. Generation of COs and GEOs from iPSCs and organoid size analysis

(A) Experimental design to generate COs and GEOs. Organoids were imaged every 30 days from 30 to 180 DIV. Organoids were harvested, and media were collected at select time points.

(B) Bright-field representative images of COs and GEOs at all time points.

(C and D) Bright-field image analysis of (C) CO and (D) GEO area relative to *3/3* (*y* axis) at each time point (*x* axis). N = 55–60 organoids from 3 *APOE3/3* and 3 *APOE4/4* iPSC lines (2 independent replicates per iPSC line pair). Data are represented as mean ± SEM. Unpaired t tests with Welch's correction were used to determine significance.

See Figure S2. $^{**}p < 0.001$, $^{***}p < 0.0005$, $^{****}p < 0.0001$, ns: not significant. Scale bars, 1 mm.

At 30 DIV, COs expressed cortical NP marker PAX6 and contained ventricular-like regions (Figure S2A) (Nieto-Estévez et al., 2022; Paşca et al., 2015). Ki67, which labels proliferative cells, was expressed in proliferative NSCs and NPs localized within ventricular-like regions (Figure S2C) (Lim et al., 2018). NPs then differentiate into immature neurons expressing beta-tubulin-III (TUJ1) (Figure S2C). Cleaved caspase-3 (AC3) antibody was used to detect cell death. Using these markers, we saw no difference in proliferation or differentiation in COs (Figures S2D–S2F). Decreased cell death was observed in *APOE4* COs at 30 DIV; however, levels of cell death were generally low in both genotypes (Figure S2G).

To determine the later effects of *APOE4* on neuronal development in COs, we examined layer II-IV marker SATB2 at 180 DIV, which we found to be decreased in *APOE4* COs (Figures 2A, 2B, and S3B). A decrease in BRN2, a marker of cortical neuron layers II/III, was observed only in AD *APOE4* paired lines (F1 and iM), suggesting a potential influence of genetic background (Figures 2A, 2C, and S3C). Increased cell death was observed at 150 DIV and 180 DIV (Figures 2A, 2D, S3A, and S3D–S3F). Notably, cell death at 180 DIV was primarily driven by iF2 and iM but was globally increased at 150 DIV, although cell death was generally low in both genotypes and time points (Figures S3E and S3F). These data indicate that cell death is not sufficient to explain cortical neuron loss in *APOE4* COs at later developmental stages.

An *APOE4*-mediated loss of upper-layer cortical neurons may result from an early shift from neurogenesis to gliogenesis. During embryonic development, astrocytes and oRG arise beginning at 3.5 months (aligning with 120 DIV in

Table 1. Human iPSC lines used to generate COs and GEOs

	Name	Sex	APOE	Age	Clinical Dx		Source	Identifier/source ID
F1	F1 3/3	F	3/3	75	control	parent	UTSA SCC	10201-5/Corriell (AG09173)
	F1 4/4	F	4/4	79	AD	parent	CIRM	CW50129FF1
iF2	F2 3/3	F	3/3	77	control	parent	EBiSC	BIONi037-A
	F2 i4/4	F	i4/4	–	–	isogenic	EBiSC	BIONi037-A-4
iM	M 4/4	M	4/4	80	AD	parent	EBiSC	UKBi011-A
	M i3/3	M	i3/3	–	–	isogenic	EBiSC	UKBi011-A-3

COs) (Gordon et al., 2021; Hansen et al., 2010). To test whether *APOE4* favors glial differentiation, we measured astrocyte and other glial-specific markers at gliogenic time points (150 and 180 DIV). At 150 and 180 DIV, *APOE4* COs showed a significant increase in GFAP, expressed in astrocytes at this time point, primarily in F1 and iF2, with no significant changes in the iM line (Figures 2E, 2F, S3A, and S3G–S3I). Astrocyte markers ALDH1L1 and SOX9 were globally increased in *APOE4* COs at 180 DIV (Figures 2E, 2G, and S3J–S3M). Additionally, HOPX^{+} oRG were significantly increased, indicating an overall increase in glia with *APOE4* at gliogenic time points (Figures 2E, 2H, and S3N). These data suggest that *APOE4* promotes gliogenesis, potentially at the expense of neurogenesis.

Astrocytes and NSCs both express high levels of ApoE, while neurons express ApoE in response to pathological conditions, albeit to a lesser extent (Kim et al., 2009). To determine the relationship between ApoE secretion and observed phenotypes, we measured secreted ApoE in media by enzyme-linked immunosorbent assay (ELISA) at all time points. At 30 DIV, ApoE secretion was below detectable limits likely due to low proportions of glia and NSCs (data not shown). ApoE was detectable at 120 DIV, the onset of gliogenesis, and all subsequent time points (150 and 180 DIV). At 150 and 180 DIV, there was a significant increase in ApoE secretion in *APOE4* COs, further suggesting an increase in glia, astrocytes and oRG, which express the highest amounts of ApoE (Figure 2I).

In the AD brain, altered cleavage of amyloid precursor protein favors cleavage to the aggregate-prone Aβ42 over the more soluble Aβ40 peptide, resulting in Aβ aggregation. Human iPSC studies in cerebral organoids and 2D cultures observe increased Aβ42/40 ratio and p-tau (Lin et al., 2018; Roher et al., 1993; Wang et al., 2018). To assess AD-related pathologies in COs, we examined Aβ by IHC, Aβ42/40 secretion by ELISA, and p-tau by IHC at 180 DIV. Aβ IHC showed a trending increase in *APOE4* COs, with significant or trending increases in female lines (Figures S3O and S3P). Secreted Aβ42/40 ratios were similarly elevated in female *APOE4* COs (Figure S3Q), while the iM lines showed a significant increase in Aβ42 and Aβ40 but a decreased Aβ42/40 ratio (Figure S3Q). No batch differences were observed (2 replicates per iPSC line). Despite robust tau expression, AD-related p-tau was undetectable at 180 DIV across multiple antibodies (data not shown).

Taken together, these data indicate that *APOE4* decreases upper-layer cortical neurons, increases cell death, and increases astrocytes and oRG at developmental time points aligning with the onset of gliogenesis. These findings suggest that *APOE4* promotes gliogenesis, potentially at the expense of neurogenesis.

APOE4 accelerates neuronal differentiation in GEOs

We observed a size deficit in GEOs across all time points; therefore, we sought to determine if *APOE4* affected NP differentiation, maturation, or cell death at early and late time points. At 30 DIV, GEOs expressed the ganglionic eminence (GE) NP marker NKX2.1, which gives rise to GABAergic interneurons. We observed a significant increase in Ki67, NKX2.1, and TUJ1 in *APOE4* GEOs (Figures 3A–3D and S4B–S4D). No difference in cell death was observed (Figures S4A, S4E, and S4F). These findings suggest that *APOE4* increases GE NP proliferation and differentiation at neurogenic time points, consistent with reports in mixed NPs (Meyer et al., 2019). Based on these findings, we hypothesized that *APOE4* may accelerate differentiation and/or maturation in GEOs at later time points. At the intermediate time point of 90 DIV, NKX2.1 remained elevated in *APOE4* GEOs, suggesting that *APOE4* influences GABAergic NP fate decisions (Figures 3E, 3F, and S4G). At 180 DIV, we observed a trending increase in GABA (Figures 3E, 3G, and S4H). Calretinin (CR), a calcium-binding protein enriched in a GABAergic neuron subtype, was significantly increased in *APOE4* GEOs at 180 DIV (Figures 3E, 3H, and S4I). Together, these findings suggest that *APOE4* may accelerate neuronal maturation in GEOs.

Of note, there was no difference in ApoE secretion in GEOs, and ApoE secretion was highly dependent on the patient line or isogenic status (Figures S4J and S4K), consistent

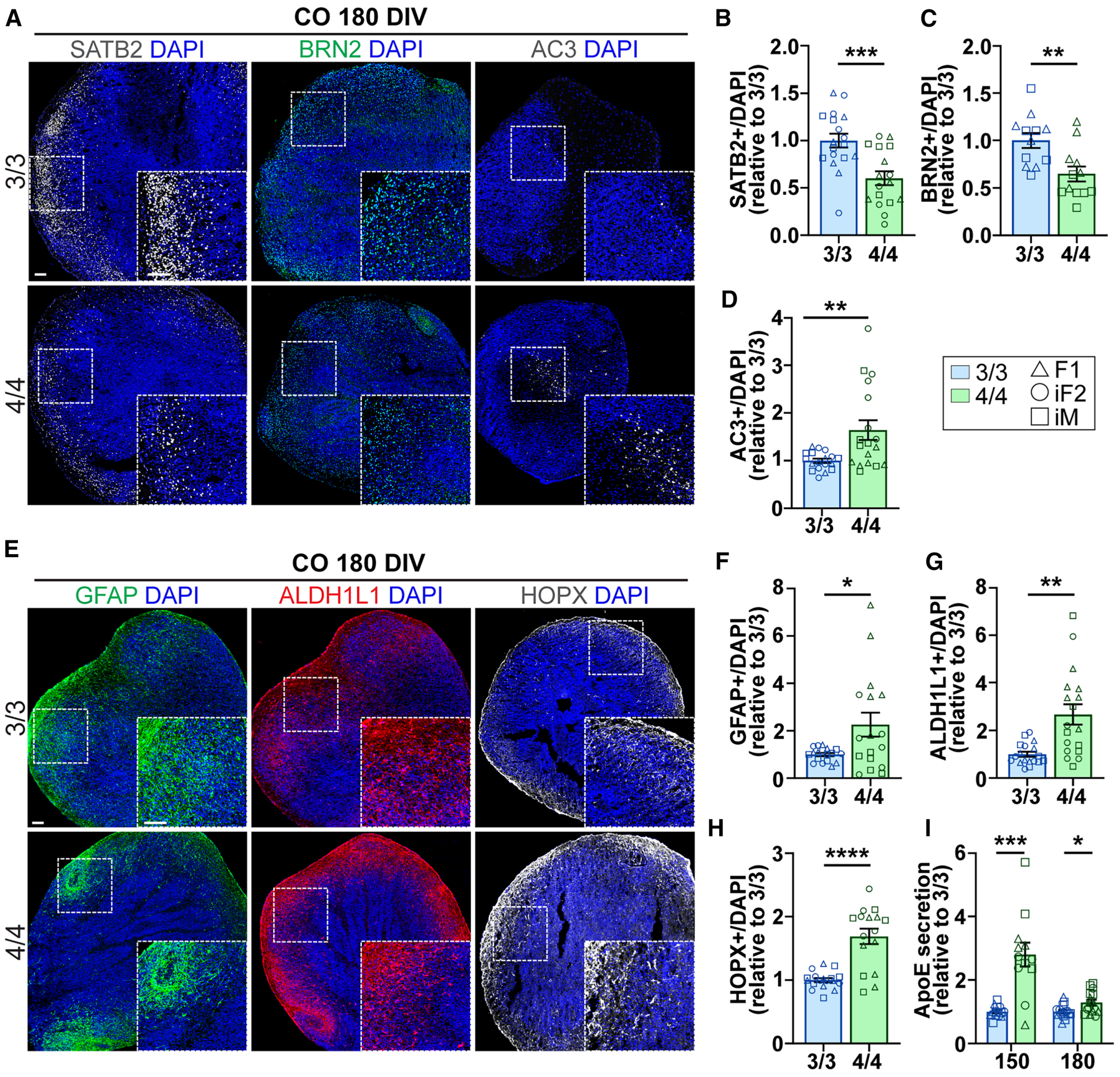

Figure 2. ***APOE4* reduces cortical neurons and increases glia at gliogenic time points**
IHC analysis and ApoE secretion in COs at gliogenic time points.
(A) IHC representative images of SATB2, BRN2, and AC3 in COs at 180 DIV.
(B–D) IHC quantification of (B) SATB2, (C) BRN2, and (D) AC3.
(E) IHC representative images of GFAP, ALDH1L1, and HOPX in COs at 180 DIV.
(F–H) IHC quantification of (F) GFAP, (G) ALDH1L1, and (H) HOPX.
(I) ApoE secretion in COs at 150 and 180 DIV relative to *APOE3/3*. IHC: bar graphs show the percentage of marker over DAPI represented relative to *APOE3/3*. $N = 16–18$ COs from 3 *APOE3/3* and 3 *APOE4/4* iPSC lines (2 replicates). BRN2, $N = 12$ organoids from F1 and iM pairs with AD *APOE4/4*. ELISA: $N = 16$ samples (each sample pooled from 3 to 4 COs) (2 replicates). Data are represented as mean ± SEM. Unpaired t tests with Welch's correction were used to determine significance.
See Figure S3. $^{*}p < 0.05$, $^{**}p < 0.01$, $^{***}p < 0.001$, $^{****}p < 0.0001$. Scale bars, 100 μm.

with reports that genetic background rather than *APOE* genotype influences ApoE expression (Tcw et al., 2022). No differences in Aβ42, Aβ40, or Aβ42/40 ratio were observed (Figures S4L–S4N). Similar to COs, AD-related p-tau was undetectable (data not shown). Taken together, these data suggest that *APOE4* affects GABAergic NPs by accelerating differentiation and maturation at early time points resulting in increased GABAergic neurons at later time points in GEOs.

APOE4 alters neurogenesis and gliogenesis transcriptional programs in COs

In COs, we observed a loss of neurons and an increase in glia at 180 DIV, suggesting that *APOE4* mediates a shift

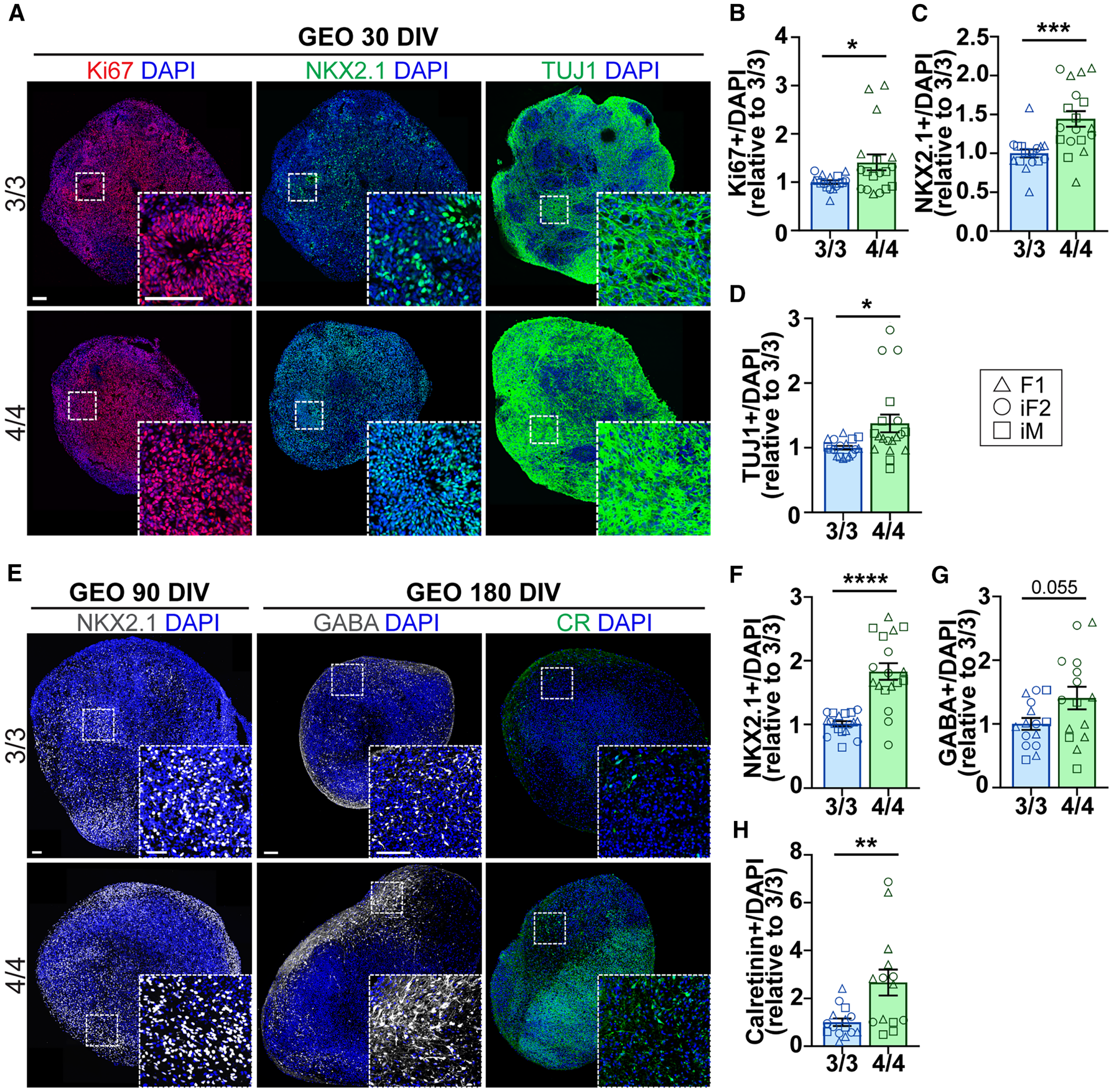

Figure 3. *APOE4* accelerates early neuronal differentiation in GEOs

IHC analysis of GEOs at 30, 90, and 180 DIV.

(A) IHC representative images of Ki67, NKX2.1, and TUJ1 in GEOs at 30 DIV.

(B–D) IHC quantification of (B) Ki67, (C) NKX2.1, and (D) TUJ1.

(E) IHC representative images of NKX2.1 at 90 DIV, GABA, and CR at 180 DIV in GEOs.

(F–H) IHC quantification of (F) NKX2.1 at 90 DIV, and (G) GABA and (H) CR at 180 DIV. IHC bar graphs show the percentage of marker to DAPI represented relative to *APOE3/3*. $N = 16–18$ organoids from 3 *APOE3/3* and 3 *APOE4/4* iPSC lines. Data are represented as mean ± SEM. Unpaired t tests with Welch's correction were used to determine significance.

See Figure S4. $^{*}p < 0.05$, $^{**}p < 0.01$, $^{***}p < 0.001$, $^{****}p < 0.0001$. Scale bars, 100 μm.

from neurogenesis to gliogenesis. To determine whether *APOE4* influences neural cell fate specification, we performed bulk RNA sequencing in COs and GEOs at neurogenic and gliogenic time points, 30 and 120 DIV, for a total of 4 conditions. For each condition, 3 organoids were pooled per sample for isogenic male and female COs and GEOs (2 replicates per line). Using t-distributed stochastic neighbor embedding analysis of all samples, organoids clustered by type and time point, confirming no batch effects and showing that each condition was transcriptomically distinct (Figure S5A). Cluster analysis revealed samples clustered by isogenic line, replicate, and genotype for each condition, indicating high batch consistency (Figures S5B–S5D). *APOE* was detectable at lower levels at

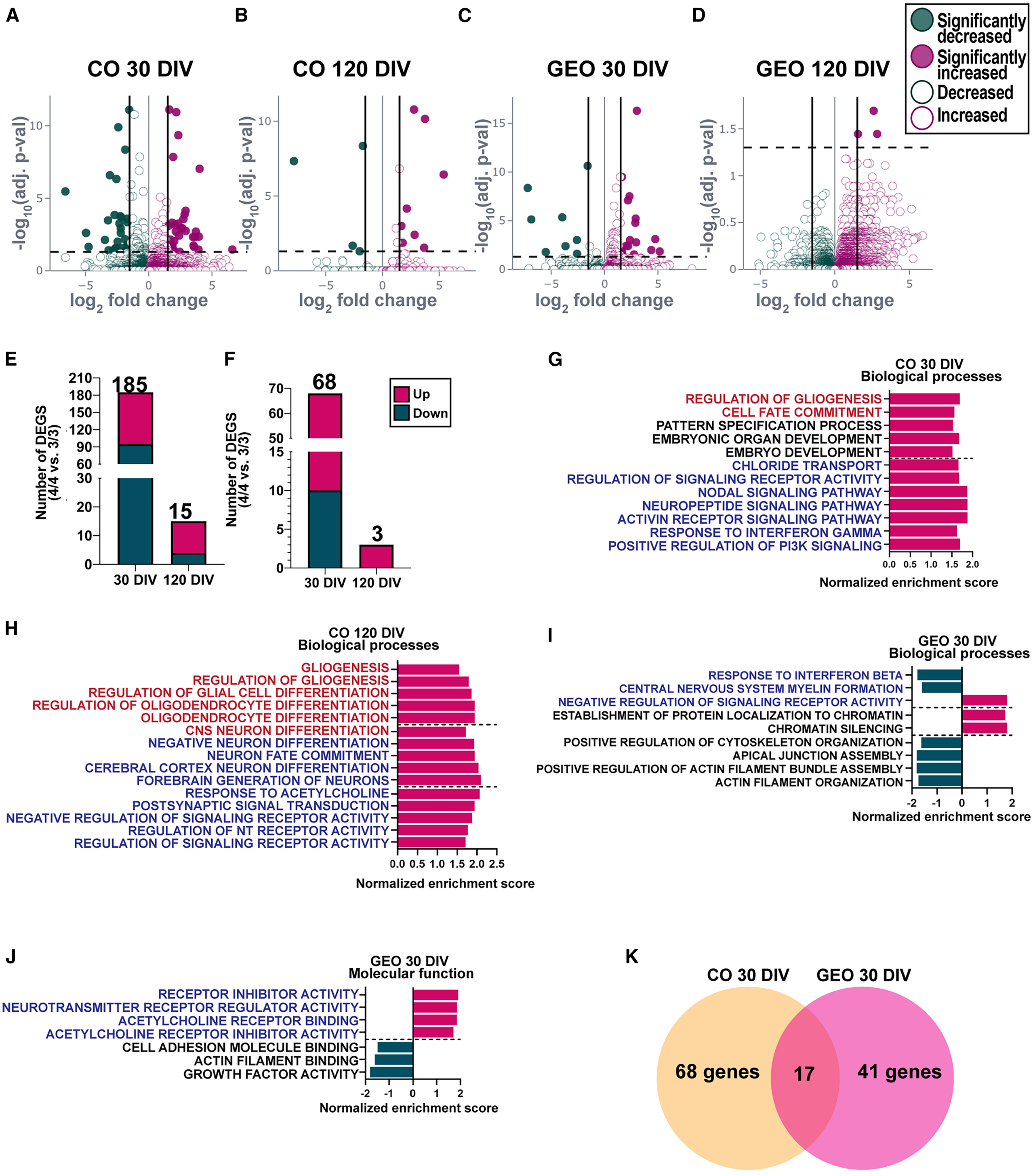

Figure 4. *APOE4* promotes gliogenesis in COs and neuron maturation in COs and GEOs

(A–D) Volcano plots of *APOE3/3* vs. *APOE4/4* in COs at (A) 30 DIV and (B) 120 DIV and GEOs at (C) 30 DIV and (D) 120 DIV. Graph shows $\log_2$ fold change (FC) (x axis) over $-\log_{10}$(adjusted p) (y axis) of genes with vertical lines at 1.2 $\log_2$(FC).

(E and F) Number of DEGs at 30 and 120 DIV in (E) COs and (F) GEOs.

(legend continued on next page)

30 DIV than at 120 DIV, consistent with our ELISA results (Figure S5E). Differential gene expression analysis comparing *APOE4* vs. *APOE3* for each condition (with sex as a covariate) identified significant differentially expressed genes (DEGs), with consistent directionality across both isogenic pairs. COs had more total DEGs than GEOs at both time points with more DEGs at 30 DIV (185 DEGs CO, 68 DEGs GEOs) than at 120 DIV (15 DEGs CO, 3 DEGs GEO), suggesting that COs are more affected by the *APOE* genotype (Figures 4A–4F). To evaluate the functional relevance of DEGs on neural cell fate, we performed fast gene set enrichment analysis and Gene Ontology (GO) using Molecular Signatures Database (MSigDB) gene sets for biological processes and molecular function.

At 30 DIV, *APOE4* COs were enriched for gene sets related to gliogenesis, development, and neuron signaling, suggesting that *APOE4* may increase neuronal differentiation and/or alter neural activity (Figure 4G). Negatively enriched gene sets included various mitochondrial and chromatin-related processes (data not shown). Consistent with 30 DIV, 120 DIV *APOE4* COs were enriched for processes related to gliogenesis, neuron differentiation, and neuron signaling (Figure 4H). Together, these results suggest that *APOE4* drives neuron differentiation, accelerates neuronal maturation, and promotes glial cell productionin COs.

In contrast, GO terms in GEOs were thematically distinct from COs. At 30 DIV, *APOE4* GEOs were enriched for biological processes related to DNA organization, cilia, and cytoskeletal organization (Figure 4I). A reduction in receptor signaling and enrichment of inhibitory receptor activity related molecular functions suggest that *APOE4* may influence inhibitory neuron maturation (Figure 4J). At 30 DIV, 17 DEGs (16 upregulated, 1 downregulated) were shared between COs and GEOs (Figure 4K). Functional annotation clustering identified 6 genes associated with cell adhesion and extracellular matrix ($p = 5.9E-5$, false discovery rate [FDR] = 7.6E−3), consistent with reports in 2D mixed neurons (Tcw et al., 2022). These genes included protocadherins, which regulate neural cell fate decisions and circuit formation during neurodevelopment (Flaherty and Maniatis, 2020). This suggests that *APOE4* drives both shared and distinct transcriptional programs in COs and GEOs, potentially leading to altered neuron function and subtype-specific vulnerability. Additionally, these results support the hypothesis that *APOE4* promotes an early shift in cell fate from neurons towards glia in COs.

APOE4 alters neural network activity patterns in fused assembloids

Heightened excitability and aberrant oscillatory synchrony observed in young *APOE4* carriers and mouse models have been linked to impaired GABAergic signaling (Najm et al., 2019). During development, intracellular chloride levels ($[Cl^-]_i$) mediate an excitatory response to GABA, which later shifts to inhibition as chloride extrusion mechanisms mature (Ben-Ari, 2002). Early expression of NKCC1, a Na^+-K^+-$2Cl^-$ cotransporter, maintains high $[Cl^-]_i$, while later expression of KCC2, a K^+-$2Cl^-$ cotransporter, lowers $[Cl^-]_i$, enabling inhibitory GABAergic signaling (Ben-Ari, 2002). In COs, but not GEOs, *APOE4* significantly reduced the expression of *GABBR1*, encoding $GABA_B$ receptor subunit 1, and *SLC12A2* and *SLC12A5*, encoding NKCC1 and KCC2, respectively (Figure S6A). GEOs showed a trend toward reduced *SLC12A2* (NKCC1) (Figure S6A). These findings suggest that *APOE4* disrupts chloride homeostasis and $GABA_B$ receptor expression in COs, potentially weakening neuronal responsiveness to GABA.

To determine whether gene expression changes related to GABA action alter neural network formation and function, we used a 3D-MEA with 60 electrodes to monitor spontaneous activity. During development, interneurons derived from GE NPs migrate into the cortex, integrating into cortical circuits (Letinic et al., 2002; Lim et al., 2018). To better recapitulate the developing neural network, we fused COs and GEOs (assembloids) at 60 DIV (Figure 5A). NPs in GEOs labeled with DLX1/2b-GFP-expressing lentivirus before fusion confirmed migration into COs at 90–120 DIV (Figure 5B). MEA recordings were performed in assembloids at 200–220 DIV in the presence or absence of GABA or the GABA receptor blocker picrotoxin (PTX). *APOE4* assembloids displayed more coordinated spiking with higher spike rates compared to *APOE3,* indicating broader connectivity (Figure 5C). Firing rates and active electrodes were slightly elevated at baseline and enhanced with PTX in *APOE4* assembloids (Figures 5D and 5E). However, since both genotypes responded similarly to PTX, these elevations are likely due to higher baseline activity in *APOE4* assembloids (Figure S6B). GABA application resulted in a stronger inhibitory response in *APOE4* assembloids, suggesting a more mature network (Figures 5E and 5F). Interestingly, baseline network

(G–J) Bar plots show GSEA for gliogenic (red), neurodevelopmental (black), and receptor activity (blue) gene sets, and normalized enrichment score represents magnitude and direction. Biological process gene sets enriched in COs at (G) 30 DIV and (H) 120 DIV and (I) GEOs at 30 DIV. (J) Molecular function gene sets in GEOs at 30 DIV.
(K) Venn diagram of overlapping DEGs in COs and GEOs at 30 DIV with p value ≤ 0.05 and 1.2 $\log_2$FC. Results are from isogenic lines (iF2 and iM), 2 independent replicates.
See Figure S5.

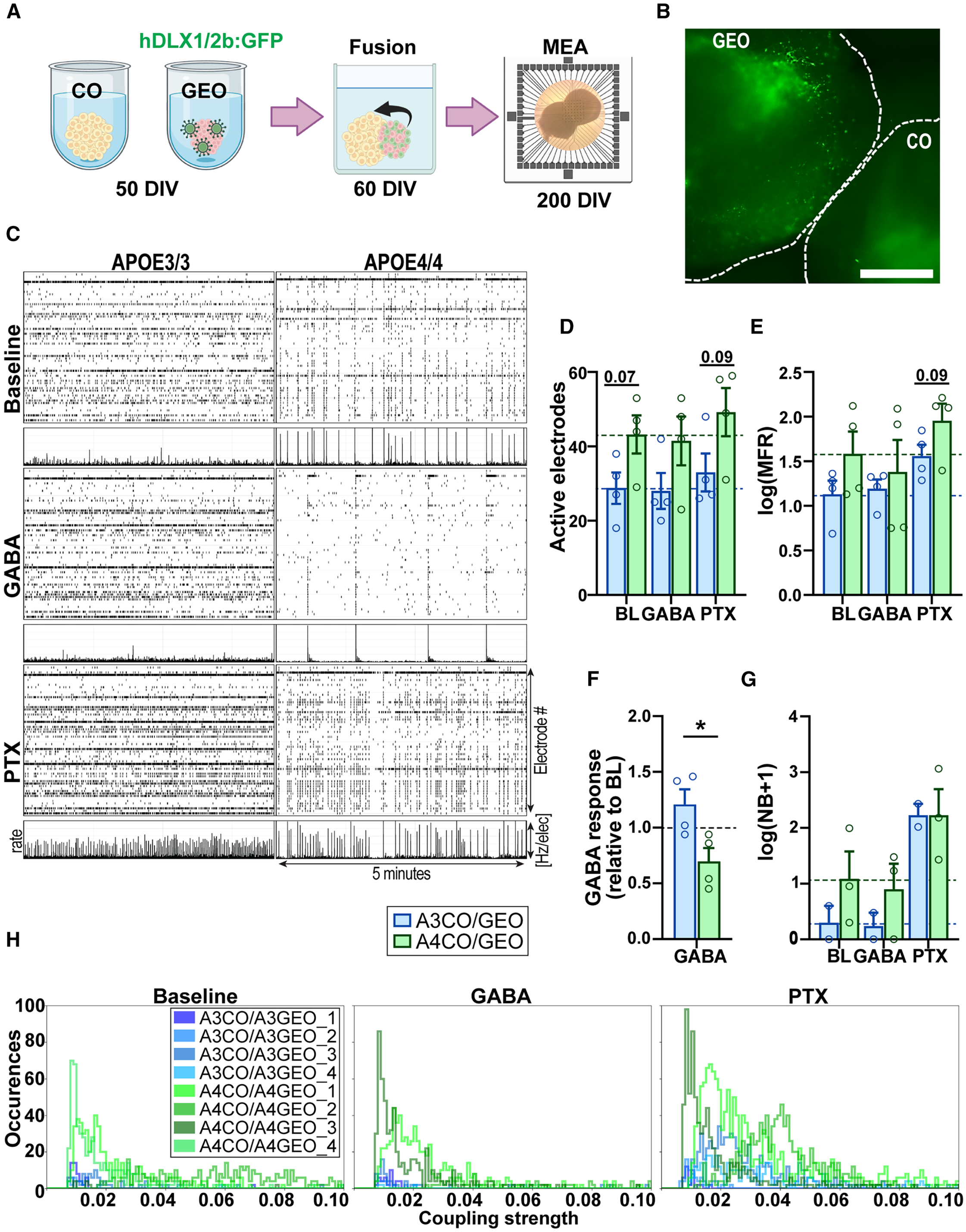

Figure 5. *APOE4* alters neural network activity patterns in assembloids

(A) Schematic of MEA experimental timeline. At 50 DIV, interneurons were labeled with hDLX1/2:GFP lentivirus in GEOs and fused at 60 DIV. 3D-MEA was performed at 200–220 DIV.

(B) Representative bright-field image of GFP-labeled GEO NPs in CO portion of assembloids.

(C) Raster plots show spikes in a representative 5 min (*x* axis) in 60 electrodes (*y* axis) from 30-min recordings at baseline (BL), GABA, and PTX with corresponding firing rate under each plot (range 0–10 Hz/s) from *APOE3* and *APOE4* assembloids.

(legend continued on next page)

bursts were observed in the majority of *APOE4* assembloids compared to only one *APOE3* assembloid, further suggesting altered neural network formation and function (Figure 5G).

During development, network synchronicity evolves into complex oscillatory activity critical for network stability and function, with GABA signaling playing a key role in this process (Puppo and Muotri, 2023). To assess network synchronicity, we measured coupling strength between electrodes using cross-correlogram analysis, which quantifies the probability of synchronous spiking between electrode pairs. *APOE4* assembloids exhibited significantly stronger synchronicity than *APOE3* in all conditions, indicating enhanced coordinated activity (Figure 5H). Despite a stronger inhibitory response to GABA, inhibition was insufficient to reduce heightened synchronicity in *APOE4* assembloids. Together, our findings suggest that *APOE4* alters GABAergic function, leading to disrupted network regulation and enhanced synchronicity.

Observed dysregulation of GABA-related genes in *APOE4* COs and heightened excitability in assembloids led us to question whether *APOE4* in COs and/or GEOs drives network excitability. To investigate this, we generated mixed-genotype assembloids by fusing *APOE3* COs with *APOE4* GEOs (*A3*CO) and *APOE4* COs with *APOE3* GEOs (*A4*CO) for MEA analysis (Figures S6C and S6D). While CO genotype significantly affected the number of active electrodes, this effect was not significant after multiple comparison corrections (Figure S6E). Similar to matched *APOE4* assembloids, *A4*CO assembloids exhibited an increased inhibitory response to GABA (Figures S6F and S6G). In contrast, *A3*CO assembloids showed a significantly greater response to PTX compared to *A4*CO assembloids (Figures S6F and S6H). Mixed assembloids exhibited more network bursts than matched *APOE4* assembloids, though to a lower rate and magnitude, with no clear genotype effect (Figure S6I). Lastly, *A4*CO assembloids displayed increased synchronicity at baseline and with GABA, similar to matched *APOE4* assembloids (Figure S6J). Together, these findings suggest that the collective changes in *APOE4* COs are sufficient to alter GABAergic function and enhance network synchrony, with broader implications for the establishment of functional neural circuits.

DISCUSSION

APOE4 induces persistent changes in brain morphology and structure in human infants that precede functional and cognitive abnormalities in adolescent and adult carriers. These studies suggest that *APOE4* triggers a cascade of compensatory events that may originate during neurodevelopment. In this study, we used regionalized neural organoids to investigate the cellular, molecular, and functional consequences of *APOE4* on cortical and GABAergic neuron development.

We report functional and region-specific consequences of *APOE4* across embryonic development (Figure 6). Our findings suggest that *APOE4* accelerates neural differentiation, disrupting the balance between neurogenesis and gliogenesis. This is supported by our observations of reduced cortical neurons and increased astrocytes and oRG in *APOE4* COs. Transcriptional enrichment for neuron maturation and gliogenic pathways further suggests an early shift in cell fate decisions in *APOE4* COs. In contrast, *APOE4* GEOs exhibited distinct yet convergent phenotypic and transcriptional changes, marked by early increases in NPs and a persistent increase in GABAergic neurons, indicative of accelerated NP differentiation. Furthermore, late-stage alterations in genes regulating GABA's inhibitory function in *APOE4* COs were associated with disrupted GABAergic signaling in assembloids. Collectively, these changes in *APOE4* COs were sufficient to alter GABA function and heighten network excitability and synchrony in both matched *APOE4* and mixed *A4*CO assembloids. Our findings provide evidence that neurodevelopmental changes driven by *APOE4* can shape early neural networks, which may subsequently contribute to AD vulnerability later in life.

APOE4 accelerates neural differentiation and gliogenesis

The loss of neurons and increase in glia in *APOE4* COs at gliogenic stages support the idea that gliogenesis occurs earlier or is favored over neurogenesis. Differential gene expression analysis revealed enrichment for gliogenesis and neuron activity gene sets, suggesting that *APOE4* may be a driver of differentiation and maturation. Our phenotypes are reminiscent of those seen in adult neurogenesis in mice, suggesting a similar mechanism in embryonic

(D–H) Bar plots show (D) active electrodes, (E) mean firing rate (MFR) log transformed, (F) GABA response (GABA over BL MFR for each assembloid), and (G) network bursts (NB) log transformed for each condition. (H) Histograms represent cross-correlation analysis of electrode coupling strength of electrode pairs (horizontal axis) and frequency of coupling strength (vertical axis). Only significantly coupled peaks are shown. $N = 4$ assembloids from *APOE3/3* and *APOE4/4* isogenic iPSC lines. To determine significance, a two-way ANOVA (Sidak's correction) was used for bar plots. Coupling strength refers to the normalized magnitude of cross-correlogram peaks, as detailed in supplemental information.
See Figure S6. $^{*}p < 0.05$, $^{**}p < 0.01$.

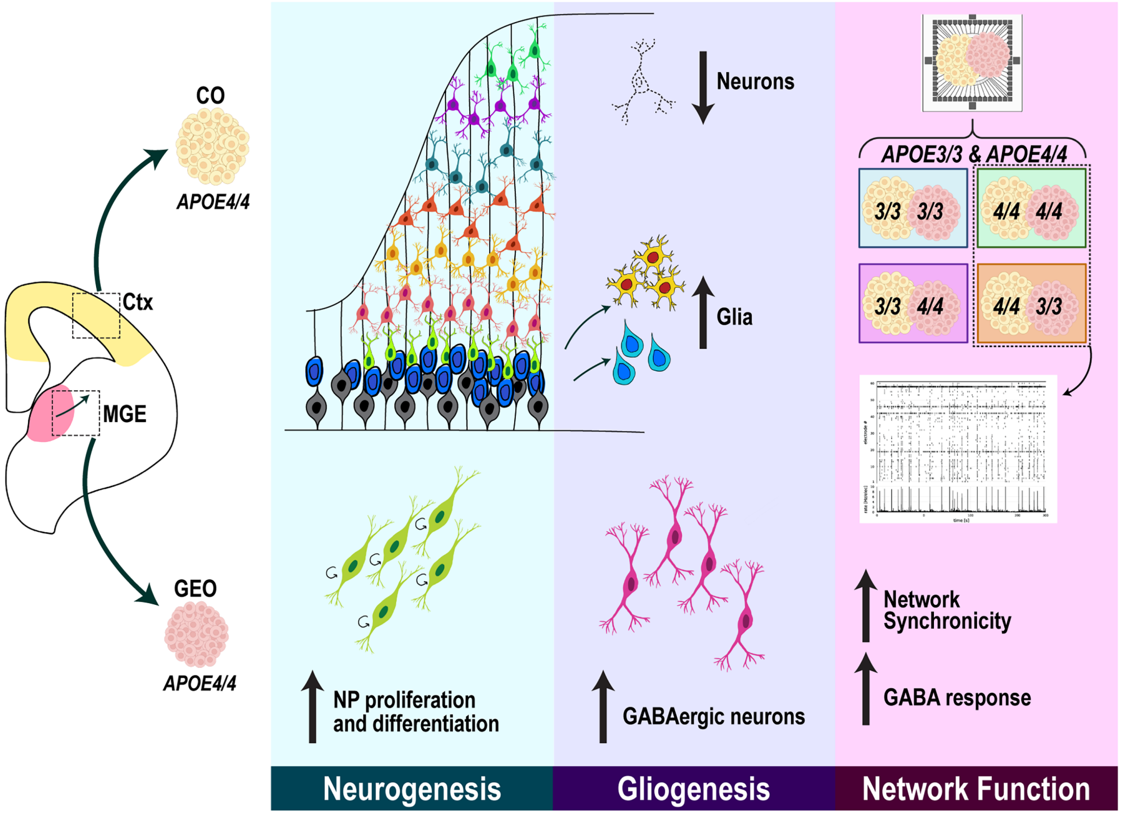

Figure 6. Summary of neurodevelopmental consequences of *APOE4*

APOE4 alters the window of neurogenesis leading to premature neuronal maturation, shifting the balance from neurogenesis to gliogenesis, contributing to altered cellular composition of neurons and glia. In GEOs, *APOE4* leads to premature GABAergic neuron maturation. Collectively, these changes are associated with heightened synchronicity and weakened inhibitory signaling in *APOE4* assembloids, with *APOE4* in COs being sufficient to drive functional changes.

neurogenesis (Li et al., 2009). Alternatively, astrocytes have been shown to regulate embryonic and adult neurogenesis and neuron circuit integration; given the reduction of neurons coinciding with gliogenesis and cell death in our study, it is possible that astrocytic *APOE4* may be the culprit (Salta et al., 2023; Watanabe et al., 2023). This is supported by no observed difference in astrocytes in GEOs.

In postmortem human brains, AD is associated with an increased number of astrocytes, a reduction of new neurons, and increased ApoE expression (Salta et al., 2023). It would be interesting to see whether *APOE4* is sufficient to alter neural cell fate dynamics in healthy postmortem brains independent of AD.

In our human iPSC model, *APOE4* dramatically increases oRG. Compared to mice, humans have oRG and differ in ApoE regulation, indicating that *APOE4* may have human-specific effects on embryonic neurogenesis. These variables could explain structural brain changes only present in adult mice. This is the first study to demonstrate that *APOE4* developmentally alters the balance of neurogenesis to gliogenesis, similar to adult neurogenesis *in vivo*, potentially corresponding to reduced GMV in *APOE4* infant cortical regions. A limitation of this organoid model is the late generation of oligodendrocytes, aligning with *in utero* generation, maturation, and myelination during later gestation and postnatal stages (Barateiro and Fernandes, 2014; Quadrato et al., 2016). While oligodendrocytes were not characterized in this study, our gene expression suggests that oligodendrocyte generation may also be accelerated. Oligodendrocyte function has been shown to be impacted by *APOE4*, although how *APOE4* impacts oligodendrocyte differentiation has not been explored (Blanchard et al., 2022). Future studies may provide insight on *APOE4*'s influence on other glial cell types and NSCs, both developmentally and in the context of AD and adult neurogenesis.

APOE4 accelerates GABAergic neuron differentiation and maturation in GEOs

Our data suggest that early increases in GABAergic NP proliferation drive later increases in differentiation and

maturation in GEOs. Throughout this study, *APOE4* GEOs were consistently smaller at all developmental stages. *APOE4* GEOs exhibited early increases in NPs, proliferation, and neurons. At later stages, *APOE4* increased GABAergic neurons, as indicated by elevated GABA and CR positivity in GEOs. Gene expression changes were most pronounced at 30 DIV, with only three differentially expressed genes at 120 DIV, suggesting a diminished impact of *APOE4* at later stages (Figure 4F).

At 30 DIV, *APOE4* GEOs showed enrichment for chromatin silencing and negative enrichment for actin/cytoskeletal organization, cell adhesion, and signaling receptor activity gene sets. Actin dynamics and cell adhesion are critical for cytoskeletal architecture, NP maintenance, proliferation, and self-renewal (Miyamoto et al., 2015). Studies in NSCs and NPs indicate that *APOE* is a transcriptional repressor and *APOE4* reduces chromatin accessibility either directly or indirectly (Jung et al., 2024; Tan et al., 2024). We also find evidence of similar mechanisms, as suggested by enrichment of chromatin silencing factors such as ZNF578, ZNF528, MEG8, and various long non-coding RNAs. These have been shown to indirectly regulate chromatin accessibility via histone modifications or miRNA expression and are implicated in neural cell fate commitment and neurological disorders (Al-Naama et al., 2020; Dong et al., 2015; Zhang et al., 2023). Further studies are needed to determine the molecular drivers of GABAergic NP fate decisions. To our knowledge, this is the first study to independently examine cortical and GABAergic neuron development and demonstrate differential *APOE4* effects.

APOE4 may induce a loss of function during development

Recent *APOE* knockout (KO) iPSC models suggest that *APOE4* may induce a developmental loss of function, with overlapping phenotypes. Single-cell RNA sequencing studies show that *APOE* KO alters neural fate in cerebral organoids, decreasing SATB2^{+} and BRN2^{+} neurons while increasing astrocytes, radial glia, and GABAergic neurons (Zhao et al., 2023). Similar findings in *APOE* KO 2D-cultured GABAergic NPs show enrichment for neuron development, function, cell adhesion, and GABAergic gene sets, along with reduced H3K27Me3, a chromatin silencing modification, in NPs and neurons. Interestingly, this could be rescued by ApoE3 at the NP stage but not the neuronal stage (Tan et al., 2024). Our study also found chromatin silencing enrichment in GEOs at 30 DIV. In adult mice, Apoe plays a role in both adult and embryonic neurogenesis, with *APOE4* and KO reducing neurogenesis and increasing gliogenesis (Yang et al., 2011). Our data suggest that *APOE4* influences neurodevelopment by reducing ApoE function, warranting further exploration of its effects on human neurogenesis and oRG.

APOE4 disrupts neural activity patterns and enhances synchronicity

APOE4 has been linked to connectivity defects, impaired GABAergic signaling, and increased network synchronicity (Najm et al., 2019). In this study, *APOE4* assembloids exhibited heightened synchronicity alongside dysregulation of $GABA_B$ receptor and Cl^- transporter gene expression. At an immature cortical stage, GABA application resulted in greater inhibition in *APOE4* assembloids, suggesting premature functional maturity of GABAergic neurons, consistent with increased GABA markers in *APOE4* GEOs.

MEA studies have shown that networks with more GABAergic neurons exhibit a stronger increase in firing when GABA is inhibited, while synchronicity depends on culture age (Sasaki et al., 2019). No genotype difference in PTX response suggests that increased synchronicity in *APOE4* assembloids results from network maturation rather than from more GABAergic neurons. Despite enhanced GABA response, GABA was not sufficient to disrupt network synchronicity, implying altered inhibitory regulation. This could be driven by disrupted chloride homeostasis and altered $GABA_B$ expression observed in *APOE4* COs. Increased NKCC1/KCC2 ratio is implicated in E/I imbalance in AD and *APOE4* (Boyarko et al., 2023). Similarly, young mice expressing human *APOE4* exhibit aberrant network oscillations stemming from altered GABAergic signaling (Chen et al., 2021).

Matched and mix-matched *APOE4* CO assembloids exhibited similar GABA responses, suggesting a CO-driven *APOE4* effect. However, mixed *A4*CO assembloids showed heightened response to PTX, possibly due to increased GABAergic neuron ratios or compensatory mechanisms modulating inhibitory balance (Sasaki et al., 2019). *APOE4*-associated hyperexcitability and inhibitory tone deficits have been linked to memory impairment in rodent models, implicating network dysfunction in memory impairment (Har-Paz et al., 2021; Nuriel et al., 2017; Peng et al., 2017). Cortical neurons treated with *APOE4* astrocyte conditioned media exhibit increased excitatory synaptic strength, implicating astrocytes in *APOE4* neuron function (Huang et al., 2019). Our findings of network abnormalities and altered GABAergic signaling align with several studies and suggest that these changes extend to neurodevelopment. Future studies should investigate whether restoring chloride homeostasis normalizes network activity and the contributions of astrocytes to inhibitory regulation with *APOE4*.

Regionalized organoids express Aβ pathology but not p-tau

We observed an increased trend in Aβ42/40 ratio in female *APOE4* COs; however, we did not detect p-tau at any time point. A previous study from our lab observed increased

Aβ42/40 and Aβ43/40 ratios in COs at 180 DIV harboring autosomal-dominant L435F PSEN1 familial AD mutation (Hurley et al., 2023). While *APOE4* cerebral organoids demonstrate p-tau and Aβ at 12 weeks, protocols similar to COs observe p-tau and Aβ only at 180 DIV, suggesting that excitatory/inhibitory neuron interactions may facilitate AD pathology (Lin et al., 2018; Zhao et al., 2020). In conclusion, further investigation in different models is needed to understand whether AD pathology is related to early *APOE4* developmental phenotypes.

Neurodevelopment is a carefully orchestrated process with critical periods that define and refine the structural and functional organization of the brain (Dehorter and Del Pino, 2020). In young *APOE4* carriers, changes in gray matter organization are accompanied by heightened network connectivity and oscillations, but whether these alterations originate during early neurodevelopment remains unclear. Here, we provide evidence that *APOE4* influences early neural circuit formation, contributing to abnormal network synchronicity. We report a nuanced role of *APOE4* in the development and patterning of excitatory and inhibitory neurons and glia, resulting in abnormal network synchronicity. Our findings suggest that early-life alterations in neural network activity may contribute to long-term functional changes in the brain. Heightened synchronicity and altered GABAergic function could drive the increased oscillatory behavior and functional hyperconnectivity observed in *APOE4* carriers. Since oscillatory network disruptions are linked to memory impairment, these early changes may render *APOE4* circuits more vulnerable to later dysfunction. Over time, increased baseline activity and altered inhibitory signaling could lead to synaptic stress and network hyperexcitability, mechanisms that are implicated in both neurodevelopmental and neurodegenerative disorders. This study is the first to explore *APOE4*'s influence on the developing human brain using brain organoids. We hope that this study will provide a foundation for further research into *APOE4*'s influence on human neurodevelopment and its relevance to AD.

METHODS

iPSC generation and characterization, and maintenance

See supplemental information for detailed characterization procedures. A total of 6 human iPSC lines were sourced for this study (Table 1). All iPSC lines had normal karyotypes (WiCell) and expressed pluripotency markers (Lin28, Nanog, Oct3/4, and SOX2). iPSCs were maintained in mTeSR 1 medium (Cat. No. 05851, STEMCELL Technologies) on 6-well tissue culture plates (Cat. No. 3506, Corning) coated with growth factor reduced Matrigel (Cat. No. 356230, BD Biosciences). Upon thawing, ROCK inhibitor Y27632 (final concentration 10 μM, Cat. No. S-1049, Selleck Chemicals) was added. Cells were passaged at 70% confluence, and Versene solution (Cat. No. 15040-066, Thermo Fisher Scientific) was used to detach cells for replating at 1:12 or frozen in knockout serum replacement (Cat No. 10828028, Gibco) and 10% DMSO.

Genotyping

APOE genotype was confirmed by Sanger sequencing (Eurofins Genomics LLC). Genomic DNA was extracted from iPSCs using DNeasy Blood & Tissue Kit (QIAprep #69504) following the manufacturer's instructions. OneTaq Hot Start DNA Polymerase (New England Biolabs, Cat. No. M0481) was used to amplify the product containing the 2 base pairs that differ between *APOE* alleles using primers (forward CTGGAGGAACAACTGACCCC, reverse CTCGAACCAGCT CTTGAGG). PCR master mix was used according to the manufacturer's instructions with the addition of 7.5% DMSO. PCR conditions were as follows: 94°C for 4 min; 94°C for 30 s, 65°C for 45 s, and 68°C for 1 min for 40 cycles; and 68°C for 5 min. PCR products (~550 bp) were visualized on an agarose gel, purified (QIAquick PCR Purification Kit Cat. No. 28106), and sent for Sanger sequencing.

CO and GEO generation

Organoids were generated using the methods described by Pasca and colleagues adapting slight modifications (Birey et al., 2017; Sloan et al., 2017). Detailed culture conditions, reagents, and media formulations can be found in supplemental methods. Each cell line pair (*APOE3/3* and *APOE4/4* isogenic pair, or *APOE3/3* control and *APOE4/4* AD patient line) was differentiated into organoids two independent times (experimental replicate), and organoids were harvested for each time point for IHC or gene expression analysis.

IHC and sample preparation

For detailed IHC conditions, table of antibodies, and additional analysis information, see supplemental methods. Four sections per organoid were imaged using a Leica (Spe8-II) or Nikon (A1R HD25) confocal microscope, and fluorescence intensity was analyzed using ImageJ software. For analysis, thresholded area of each marker was divided over thresholded DAPI for nuclear markers, or organoid area for cytoplasmic markers. Four sections were analyzed per organoid with a total of 3 organoids per replicate (2 replicates per iPSC line pair, 3 iPSC line pairs, $N = 18$ organoids). Data are represented relative to *APOE3/3* for all IHC.

RNA isolation

Organoids (3 pooled) were harvested, and RNA was extracted using the QIAGEN miRNeasy Mini Kit (Cat. No.

217004) according to manufacturer's instructions for all gene expression experiments. The concentration and purity of the RNA samples were measured by using Nanodrop (Thermo Fisher Scientific). The extracted RNA (500 ng) was reverse transcribed according to the protocol supplied with the SuperScript III First-Strand Synthesis System for reverse transcription (Invitrogen, Cat. No. 18080-051). RNA integrity number values were assessed to ensure cDNA library purity and quality.

Reverse transcriptase reaction and quantitative PCR assay

Quantitative real-time PCR was carried out in a QuantStudio5 real-time PCR system using the PowerUp SYBR Green Master Mix methodology following the manufacturer's instructions (Applied Biosystems, Cat. No. A25742). Reactions were run in triplicate, and the expression of each gene was normalized to the geometric mean of GAPDH as a housekeeping gene and analyzed by using the ΔΔCT method. The primer sequences of each gene are listed in Table S2.

Bulk RNA sequencing analysis

RNA sequencing library was generated using NEBNext Ultra II Directional RNA kit (Cat No. E7765, NEB) according to manufacturer's instructions. 75 paired-end sequencing was performed by the UTSA Genomics Core, as detailed in supplemental methods. The samples sequenced were from four conditions: COs and GEOs at 30 DIV and 120 DIV from isogenic pairs (iF2 and iM), and 2 replicates per iPSC line, for a total of 32 samples, all sequenced at the same time. Data were processed and analyzed using Pluto (pluto.bio) as detailed in supplemental methods. Prior to differential gene expression analysis, genes were prefiltered to exclude genes with less than 3 reads in 20% of samples in any group. Differential expression analysis was performed with the DESeq2 R package (Love et al., 2014) comparing *APOE3/3* vs. *APOE4/4* for each condition with sex as a covariate. Log_2 fold change was calculated for each comparison. FDR was used to correct for multiple testing (Love et al., 2014). Adjusted p value of <0.05 was considered significant. Gene set enrichment analysis (GSEA) was performed with DEGs ranked by $\log_2$ fold change. Gene sets from MSigDB, biological process and molecular function gene sets, were curated (prefiltered to 5–1,000 genes) using the msigdbr R package. See Table S3 for software packages and analysis details.

Measurement of ApoE, $A\beta_{40}$, and $A\beta_{42}$ in the medium from organoids

At selected time points, the medium was collected from organoid cultures (3–4 organoids after 3–4 days in media) and stored at −80°C. Aβ peptides were measured with Human β Amyloid (1–42) ELISA kit (Wako Chemicals, Cat. No 298-624-01) with undiluted media and Wako Human β Amyloid (1–40) ELISA kit (Wako Chemicals, Cat. No 298-64601) with 1:4 diluted media. Secreted ApoE was measured with Apolipoprotein E Human ELISA Kit (Thermo Fisher Scientific, Cat. No. EHAPOE) with 1:3 diluted media. Plates were measured with GloMax Explorer Multimode Microplate reader (GM3500), and data were analyzed using GraphPad Prism 9 software.

MEA recording and analysis

Detailed protocol, equipment, and analysis used can be found in supplemental methods. All MEA experiments were performed on 200 DIV organoids using 3D-MEA (60-3DMEA200/12/80iR-Ti, Multichannel system, Harvard Bioscience) chips. Sequential recordings were performed as follows: baseline, 20 μM GABA (56-12-2, Sigma-Aldrich), and 58 μM PTX (124-87-8, Sigma-Aldrich), for 30 min each condition, with recording starting 10 min after drug application. Raw electrical potentials were amplified using an electronic amplifier (ME2100-Mini, Multichannel systems, Harvard Bioscience), sampled at 25 kHz/channel, and digitized at 16-bit resolution.

Statistical analysis

With the exception of coupling strength analysis, all analyses were carried out with GraphPad Prism software, and the differences were considered statistically significant when $p < 0.05$. Outliers were removed from each dataset using GraphPad Prism software (Q = 1%). A two-tailed unpaired Student's t test was used to compare the mean ± standard error of the mean (SEM) values, with Welch's correction when the F-test indicated significant differences between the variances of both groups. For data shown as relative to control, all values were normalized to the average of the control for each iPSC line pair. Organoid size: Holm-Šídák correction for multiple comparisons was performed. MEA: for baseline spike count and active electrodes, a two-tailed unpaired t test was used to determine significance. A paired t test was used to compare the effect of chemical application and genotype on active electrodes and spike count at baseline. Coupling strength analysis was performed using MATLAB (see supplemental methods for details), and a Kruskal-Wallis rank-sum test with chi-square approximation was used to determine significance between *APOE3/3* and *APOE4/4*, or mixed *A3*CO and *A4*CO, at baseline, GABA, and PTX application.

RESOURCE AVAILABILITY

Lead contact

Requests for further information and resources should be directed to and will be fulfilled by the lead contact, Jenny Hsieh (jenny.hsieh@utsa.edu).

Materials availability

iPSC line 10201-5 was generated and characterized by the UTSA SCC. It is available on request with executed material transfer agreement. Any information and requests for this line should be directed to christopher.navara@utsa.edu.

Data and code availability

The accession number for RNA-seq data generated in this study is GSE289912. MEA analysis codes can be found at https://github.com/mgiugliano/SPiQ.

ACKNOWLEDGMENTS

This work was supported by NIH grants (U01DA054170, R01NS113516, R01NS124855, and R21AG066496), Robert J. Kleberg, Jr. and Helen C. Kleberg Foundation, and the Semmes Foundation (to J.H.); and NIH 1F31AG082498 (to K.K.M.-A.). We would like to thank Bess Frost, Hyoung-gon Lee, and Chris Gamblin for their help with reagents, protocols, and advice on the project. Some figures were created with BioRender.com. Some plots and analysis were performed using Pluto (https://pluto.bio).

AUTHOR CONTRIBUTIONS

Conceptualization, K.K.M.-A., J.H., and V.N.-E.; methodology, K.K.M.-A., E.D.-G., P.V., and J.H.; software, A.H.; formal analysis, K.K.M.-A., E.D.-G., S.M., and A.H.; investigation, K.K.M.-A., E.D.-G., A.A., S.M., A.M.-P., and Y.R.; resources, C.N.; writing – original draft, K.K.M.-A.; writing – review and editing, K.K.M.-A., E.D.G., P.V., S.M., A.M.-P., V.N.-E., and J.H.; visualization, K.K.M.-A. and A.H.; supervision, K.K.M.-A., M.G., and J.H.; funding acquisition, K.K.M.-A. and J.H.

DECLARATION OF INTERESTS

The authors declare no competing interests.

DECLARATION OF GENERATIVE AI AND AI-ASSISTED TECHNOLOGIES IN THE WRITING PROCESS

During the editing process, the authors used ChatGPT to reduce word count and redundancy without changing the manuscript structure or content. All minor changes were curated by the authors, and we take full responsibility for the content of the publication.

SUPPLEMENTAL INFORMATION

Supplemental information can be found online at https://doi.org/10.1016/j.stemcr.2025.102537.

Received: June 5, 2024
Revised: May 20, 2025
Accepted: May 21, 2025
Published: June 19, 2025

REFERENCES

Al-Naama, N., Mackeh, R., and Kino, T. (2020). C(2)H(2)-Type Zinc Finger Proteins in Brain Development, Neurodevelopmental, and Other Neuropsychiatric Disorders: Systematic Literature-Based Analysis. Front. Neurol. *11*, 32. https://doi.org/10.3389/fneur.2020.00032.

Area-Gomez, E., Larrea, D., Pera, M., Agrawal, R.R., Guilfoyle, D.N., Pirhaji, L., Shannon, K., Arain, H.A., Ashok, A., Chen, Q., et al. (2020). *APOE4* is Associated with Differential Regional Vulnerability to Bioenergetic Deficits in Aged APOE Mice. Sci. Rep. *10*, 4277. https://doi.org/10.1038/s41598-020-61142-8.

Baig, S., Nadaf, J., Allache, R., Le, P.U., Luo, M., Djedid, A., Nkili-Meyong, A., Safisamghabadi, M., Prat, A., Antel, J., et al. (2024). Identity and nature of neural stem cells in the adult human subventricular zone. iScience *27*, 109342. https://doi.org/10.1016/j.isci.2024.109342.

Barateiro, A., and Fernandes, A. (2014). Temporal oligodendrocyte lineage progression: in vitro models of proliferation, differentiation and myelination. Biochim Biophys Acta *1843*, 1917–1929. https://doi.org/10.1016/j.bbamcr.2014.04.018.

Ben-Ari, Y. (2002). Excitatory actions of gaba during development: the nature of the nurture. Nat. Rev. Neurosci. *3*, 728–739. https://doi.org/10.1038/nrn920.

Birey, F., Andersen, J., Makinson, C.D., Islam, S., Wei, W., Huber, N., Fan, H.C., Metzler, K.R.C., Panagiotakos, G., Thom, N., et al. (2017). Assembly of functionally integrated human forebrain spheroids. Nature *545*, 54–59. https://doi.org/10.1038/nature22330.

Blanchard, J.W., Akay, L.A., Davila-Velderrain, J., von Maydell, D., Mathys, H., Davidson, S.M., Effenberger, A., Chen, C.-Y., Maner-Smith, K., Hajjar, I., et al. (2022). APOE4 impairs myelination via cholesterol dysregulation in oligodendrocytes. Nature *611*, 769–779. https://doi.org/10.1038/s41586-022-05439-w.

Boyarko, B., Podvin, S., Greenberg, B., Momper, J.D., Huang, Y., Gerwick, W.H., Bang, A.G., Quinti, L., Griciuc, A., Kim, D.Y., et al. (2023). Evaluation of bumetanide as a potential therapeutic agent for Alzheimer's disease. Front. Pharmacol. *14*, 1190402.

Cacciaglia, R., Molinuevo, J.L., Falcón, C., Arenaza-Urquijo, E.M., Sánchez-Benavides, G., Brugulat-Serrat, A., Blennow, K., Zetterberg, H., and Gispert, J.D.; ALFA study (2020). APOE-ε4 Shapes the Cerebral Organization in Cognitively Intact Individuals as Reflected by Structural Gray Matter Networks. Cereb. Cortex *30*, 4110–4120. https://doi.org/10.1093/cercor/bhaa034.

Chang, L., Douet, V., Bloss, C., Lee, K., Pritchett, A., Jernigan, T.L., Akshoomoff, N., Murray, S.S., Frazier, J., Kennedy, D.N., et al. (2016). Gray matter maturation and cognition in children with different APOE ε genotypes. Neurology *87*, 585–594. https://doi.org/10.1212/wnl.0000000000002939.

Chen, M., Chen, Y., Huo, Q., Wang, L., Tan, S., Misrani, A., Jiang, J., Chen, J., Chen, S., Zhang, J., et al. (2021). Enhancing GABAergic signaling ameliorates aberrant gamma oscillations of olfactory bulb in AD mouse models. Mol. Neurodegener. *16*, 14. https://doi.org/10.1186/s13024-021-00434-7.

Dean, D.C., 3rd, Jerskey, B.A., Chen, K., Protas, H., Thiyyagura, P., Roontiva, A., O'Muircheartaigh, J., Dirks, H., Waskiewicz, N., Lehman, K., et al. (2014). Brain differences in infants at differential genetic risk for late-onset Alzheimer disease: a cross-sectional imaging study. JAMA Neurol. *71*, 11–22. https://doi.org/10.1001/jamaneurol.2013.4544.

Dehorter, N., and Del Pino, I. (2020). Shifting Developmental Trajectories During Critical Periods of Brain Formation. Front. Cell. Neurosci. *14*, 283. https://doi.org/10.3389/fncel.2020.00283.

Dong, X., Chen, K., Cuevas-Diaz Duran, R., You, Y., Sloan, S.A., Zhang, Y., Zong, S., Cao, Q., Barres, B.A., and Wu, J.Q. (2015). Comprehensive Identification of Long Non-coding RNAs in Purified Cell Types from the Brain Reveals Functional LncRNA in OPC Fate Determination. PLoS Genet. *11*, e1005669. https://doi.org/10.1371/journal.pgen.1005669.

Filippini, N., MacIntosh, B.J., Hough, M.G., Goodwin, G.M., Frisoni, G.B., Smith, S.M., Matthews, P.M., Beckmann, C.F., and Mackay, C.E. (2009). Distinct patterns of brain activity in young carriers of the *APOE*-ε4 allele. Proc. Natl. Acad. Sci. USA *106*, 7209–7214. https://doi.org/10.1073/pnas.0811879106.

Flaherty, E., and Maniatis, T. (2020). The role of clustered protocadherins in neurodevelopment and neuropsychiatric diseases. Curr. Opin. Genet. Dev. *65*, 144–150. https://doi.org/10.1016/j.gde.2020.05.041.

Gillespie, A.K., Jones, E.A., Lin, Y.H., Karlsson, M.P., Kay, K., Yoon, S.Y., Tong, L.M., Nova, P., Carr, J.S., Frank, L.M., and Huang, Y. (2016). Apolipoprotein E4 Causes Age-Dependent Disruption of Slow Gamma Oscillations during Hippocampal Sharp-Wave Ripples. Neuron *90*, 740–751. https://doi.org/10.1016/j.neuron.2016.04.009.

Gordon, A., Yoon, S.-J., Tran, S.S., Makinson, C.D., Park, J.Y., Andersen, J., Valencia, A.M., Horvath, S., Xiao, X., Huguenard, J.R., et al. (2021). Long-term maturation of human cortical organoids matches key early postnatal transitions. Nat. Neurosci. *24*, 331–342. https://doi.org/10.1038/s41593-021-00802-y.

Hansen, D.V., Lui, J.H., Parker, P.R.L., and Kriegstein, A.R. (2010). Neurogenic radial glia in the outer subventricular zone of human neocortex. Nature *464*, 554–561. https://doi.org/10.1038/nature08845.

Har-Paz, I., Arieli, E., and Moran, A. (2021). *APOE4* attenuates cortical neuronal activity in young behaving *APOE4* rats. Neurobiol. Dis. *155*, 105373. https://doi.org/10.1016/j.nbd.2021.105373.

Harman, D. (2006). Alzheimer's disease pathogenesis: role of aging. Ann. N. Y. Acad. Sci. *1067*, 454–460. https://doi.org/10.1196/annals.1354.065.

Holtzman, D.M., Morris, J.C., and Goate, A.M. (2011). Alzheimer's disease: the challenge of the second century. Sci. Transl. Med. *3*, 77sr71. https://doi.org/10.1126/scitranslmed.3002369.

Huang, Y.-W.A., Zhou, B., Nabet, A.M., Wernig, M., and Südhof, T. C. (2019). Differential Signaling Mediated by ApoE2, ApoE3, and *APOE4* in Human Neurons Parallels Alzheimer's Disease Risk. J. Neurosci. *39*, 7408–7427. https://doi.org/10.1523/jneurosci.2994-18.2019.

Hurley, E.M., Mozolewski, P., Dobrowolski, R., and Hsieh, J. (2023). Familial Alzheimer's disease-associated PSEN1 mutations affect neurodevelopment through increased Notch signaling. Stem Cell Rep. *18*, 1516–1533. https://doi.org/10.1016/j.stemcr.2023.05.018.

Jung, M., Jung, J.-S., Pfeifer, J., Hartmann, C., Ehrhardt, T., Abid, C. L., Kintzel, J., Puls, A., Navarrete Santos, A., Hollemann, T., et al. (2024). Neuronal Stem Cells from Late-Onset Alzheimer Patients Show Altered Regulation of Sirtuin 1 Depending on Apolipoprotein E Indicating Disturbed Stem Cell Plasticity. Mol. Neurobiol. *61*, 1562–1579. https://doi.org/10.1007/s12035-023-03633-z.

Karch, C.M., Cruchaga, C., and Goate, A.M. (2014). Alzheimer's disease genetics: from the bench to the clinic. Neuron *83*, 11–26. https://doi.org/10.1016/j.neuron.2014.05.041.

Kim, J., Basak, J.M., and Holtzman, D.M. (2009). The role of apolipoprotein E in Alzheimer's disease. Neuron *63*, 287–303. https://doi.org/10.1016/j.neuron.2009.06.026.

Knickmeyer, R.C., Wang, J., Zhu, H., Geng, X., Woolson, S., Hamer, R.M., Konneker, T., Lin, W., Styner, M., and Gilmore, J.H. (2014). Common variants in psychiatric risk genes predict brain structure at birth. Cereb. Cortex *24*, 1230–1246. https://doi.org/10.1093/cercor/bhs401.

Knoferle, J., Yoon, S.Y., Walker, D., Leung, L., Gillespie, A.K., Tong, L.M., Bien-Ly, N., and Huang, Y. (2014). Apolipoprotein E4 produced in GABAergic interneurons causes learning and memory deficits in mice. J. Neurosci. *34*, 14069–14078. https://doi.org/10.1523/jneurosci.2281-14.2014.

Letinic, K., Zoncu, R., and Rakic, P. (2002). Origin of GABAergic neurons in the human neocortex. Nature *417*, 645–649. https://doi.org/10.1038/nature00779.

Li, G., Bien-Ly, N., Andrews-Zwilling, Y., Xu, Q., Bernardo, A., Ring, K., Halabisky, B., Deng, C., Mahley, R.W., and Huang, Y. (2009). GABAergic interneuron dysfunction impairs hippocampal neurogenesis in adult apolipoprotein E4 knockin mice. Cell Stem Cell *5*, 634–645. https://doi.org/10.1016/j.stem.2009.10.015.

Lim, L., Mi, D., Llorca, A., and Marín, O. (2018). Development and Functional Diversification of Cortical Interneurons. Neuron *100*, 294–313. https://doi.org/10.1016/j.neuron.2018.10.009.

Lin, Y.T., Seo, J., Gao, F., Feldman, H.M., Wen, H.L., Penney, J., Cam, H.P., Gjoneska, E., Raja, W.K., Cheng, J., et al. (2018). *APOE4* Causes Widespread Molecular and Cellular Alterations Associated with Alzheimer's Disease Phenotypes in Human iPSC-Derived Brain Cell Types. Neuron *98*, 1141–1154.e7. https://doi.org/10.1016/j.neuron.2018.05.008.

Love, M.I., Huber, W., and Anders, S. (2014). Moderated estimation of fold change and dispersion for RNA-seq data with DESeq2. Genome Biol. *15*, 550. https://doi.org/10.1186/s13059-014-0550-8.

Maloney, B., Ge, Y.W., Alley, G.M., and Lahiri, D.K. (2007). Important differences between human and mouse APOE gene promoters: limitation of mouse APOE model in studying Alzheimer's disease. J. Neurochem. *103*, 1237–1257. https://doi.org/10.1111/j.1471-4159.2007.04831.x.

Meyer, K., Feldman, H.M., Lu, T., Drake, D., Lim, E.T., Ling, K.H., Bishop, N.A., Pan, Y., Seo, J., Lin, Y.T., et al. (2019). REST and Neural Gene Network Dysregulation in iPSC Models of Alzheimer's Disease. Cell Rep. *26*, 1112–1127.e9. https://doi.org/10.1016/j.celrep.2019.01.023.

Miyamoto, Y., Sakane, F., and Hashimoto, K. (2015). N-cadherin-based adherens junction regulates the maintenance, proliferation, and differentiation of neural progenitor cells during development.

Cell Adh. Migr. *9*, 183–192. https://doi.org/10.1080/19336918.2015.1005466.

Najm, R., Jones, E.A., and Huang, Y. (2019). Apolipoprotein E4, inhibitory network dysfunction, and Alzheimer's disease. Mol. Neurodegener. *14*, 24. https://doi.org/10.1186/s13024-019-0324-6.

Nieto-Estévez, V., Donegan, J.J., McMahon, C.L., Elam, H.B., Chavera, T.A., Varma, P., Berg, K.A., Lodge, D.J., and Hsieh, J. (2022). Buprenorphine Exposure Alters the Development and Migration of Interneurons in the Cortex. Front. Mol. Neurosci. *15*, 889922. https://doi.org/10.3389/fnmol.2022.889922.

Nimsanor, N., Jørring, I., Rasmussen, M.A., Clausen, C., Mau-Holzmann, U.A., Bus, C., Hoffmann, S.A., Gasser, T., Kluba, T., Holst, B., and Schmid, B. (2016). Generation of induced pluripotent stem cells derived from a 77-year-old healthy woman as control for age related diseases. Stem Cell Res. *17*, 550–552. https://doi.org/10.1016/j.scr.2016.09.019.

Nuriel, T., Angulo, S.L., Khan, U., Ashok, A., Chen, Q., Figueroa, H. Y., Emrani, S., Liu, L., Herman, M., Barrett, G., et al. (2017). Neuronal hyperactivity due to loss of inhibitory tone in *APOE4* mice lacking Alzheimer's disease-like pathology. Nat. Commun. *8*, 1464. https://doi.org/10.1038/s41467-017-01444-0.

O'Dwyer, L., Lamberton, F., Matura, S., Tanner, C., Scheibe, M., Miller, J., Rujescu, D., Prvulovic, D., and Hampel, H. (2012). Reduced hippocampal volume in healthy young *APOE4* carriers: an MRI study. PLoS One *7*, e48895. https://doi.org/10.1371/journal.pone.0048895.

Paşca, A.M., Sloan, S.A., Clarke, L.E., Tian, Y., Makinson, C.D., Huber, N., Kim, C.H., Park, J.Y., O'Rourke, N.A., Nguyen, K.D., et al. (2015). Functional cortical neurons and astrocytes from human pluripotent stem cells in 3D culture. Nat. Methods *12*, 671–678. https://doi.org/10.1038/nmeth.3415.

Peitz, M., Bechler, T., Thiele, C.C., Veltel, M., Bloschies, M., Fliessbach, K., Ramirez, A., and Brüstle, O. (2018). Blood-derived integration-free iPS cell line UKBi011-A from a diagnosed male Alzheimer's disease patient with APOE ϵ4/ϵ4 genotype. Stem Cell Res. *29*, 250–253. https://doi.org/10.1016/j.scr.2018.04.011.

Peng, K.Y., Mathews, P.M., Levy, E., and Wilson, D.A. (2017). Apolipoprotein E4 causes early olfactory network abnormalities and short-term olfactory memory impairments. Neuroscience *343*, 364–371. https://doi.org/10.1016/j.neuroscience.2016.12.004.

Puppo, F., and Muotri, A.R. (2023). Network and Microcircuitry Development in Human Brain Organoids. Biol. Psychiatry *93*, 590–593. https://doi.org/10.1016/j.biopsych.2022.07.006.

Quadrato, G., Brown, J., and Arlotta, P. (2016). The promises and challenges of human brain organoids as models of neuropsychiatric disease. Nat Med *22*, 1220–1228. https://doi.org/10.1038/nm.4214.

Rakic, P. (2009). Evolution of the neocortex: a perspective from developmental biology. Nat. Rev. Neurosci. *10*, 724–735. https://doi.org/10.1038/nrn2719.

Remer, J., Dean, D.C., 3rd, Chen, K., Reiman, R.A., Huentelman, M. J., Reiman, E.M., and Deoni, S.C.L. (2020). Longitudinal white matter and cognitive development in pediatric carriers of the apolipoprotein ε4 allele. Neuroimage *222*, 117243. https://doi.org/10.1016/j.neuroimage.2020.117243.

Reynolds, C.A., Smolen, A., Corley, R.P., Munoz, E., Friedman, N.P., Rhee, S.H., Stallings, M.C., DeFries, J.C., and Wadsworth, S.J. (2019). APOE effects on cognition from childhood to adolescence. Neurobiol. Aging *84*, 239.e1–239.e8. https://doi.org/10.1016/j.neurobiolaging.2019.04.011.

Roher, A.E., Lowenson, J.D., Clarke, S., Woods, A.S., Cotter, R.J., Gowing, E., and Ball, M.J. (1993). beta-Amyloid-(1-42) is a major component of cerebrovascular amyloid deposits: implications for the pathology of Alzheimer disease. Proc. Natl. Acad. Sci. USA *90*, 10836–10840. https://doi.org/10.1073/pnas.90.22.10836.

Salta, E., Lazarov, O., Fitzsimons, C.P., Tanzi, R., Lucassen, P.J., and Choi, S.H. (2023). Adult hippocampal neurogenesis in Alzheimer's disease: A roadmap to clinical relevance. Cell Stem Cell *30*, 120–136. https://doi.org/10.1016/j.stem.2023.01.002.

Sasaki, T., Suzuki, I., Yokoi, R., Sato, K., and Ikegaya, Y. (2019). Synchronous spike patterns in differently mixed cultures of human iPSC-derived glutamatergic and GABAergic neurons. Biochem. Biophys. Res. Commun. *513*, 300–305. https://doi.org/10.1016/j.bbrc.2019.03.161.

Shaw, P., Lerch, J.P., Pruessner, J.C., Taylor, K.N., Rose, A.B., Greenstein, D., Clasen, L., Evans, A., Rapoport, J.L., and Giedd, J.N. (2007). Cortical morphology in children and adolescents with different apolipoprotein E gene polymorphisms: an observational study. Lancet Neurol. *6*, 494–500. https://doi.org/10.1016/s1474-4422(07)70106-0.

Sloan, S.A., Darmanis, S., Huber, N., Khan, T.A., Birey, F., Caneda, C., Reimer, R., Quake, S.R., Barres, B.A., and Paşca, S.P. (2017). Human Astrocyte Maturation Captured in 3D Cerebral Cortical Spheroids Derived from Pluripotent Stem Cells. Neuron *95*, 779–790.e6. https://doi.org/10.1016/j.neuron.2017.07.035.

Tan, J., Tan, Y.Y., Ngian, Z.K., Chong, S.Y., Rao, V.K., Wang, J.W., Zeng, X., and Ong, C.T. (2024). ApoE maintains neuronal integrity via microRNA and H3K27me3-mediated repression. iScience *27*, 109231. https://doi.org/10.1016/j.isci.2024.109231.

Tcw, J., Qian, L., Pipalia, N.H., Chao, M.J., Liang, S.A., Shi, Y., Jain, B.R., Bertelsen, S.E., Kapoor, M., Marcora, E., et al. (2022). Cholesterol and matrisome pathways dysregulated in astrocytes and microglia. Cell *185*, 2213–2233.e25. https://doi.org/10.1016/j.cell.2022.05.017.

Tong, L.M., Yoon, S.Y., Andrews-Zwilling, Y., Yang, A., Lin, V., Lei, H., and Huang, Y. (2016). Enhancing GABA Signaling during Middle Adulthood Prevents Age-Dependent GABAergic Interneuron Decline and Learning and Memory Deficits in *APOE4* Mice. J. Neurosci. *36*, 2316–2322. https://doi.org/10.1523/JNEUROSCI.3815-15.2016.

Wang, C., Najm, R., Xu, Q., Jeong, D.E., Walker, D., Balestra, M.E., Yoon, S.Y., Yuan, H., Li, G., Miller, Z.A., et al. (2018). Gain of toxic apolipoprotein E4 effects in human iPSC-derived neurons is ameliorated by a small-molecule structure corrector. Nat. Med. *24*, 647–657. https://doi.org/10.1038/s41591-018-0004-z.

Watanabe, H., Murakami, R., Tsumagari, K., Morimoto, S., Hashimoto, T., Imaizumi, K., Sonn, I., Yamada, K., Saito, Y., Murayama, S., et al. (2023). Astrocytic *APOE4* genotype-mediated negative impacts on synaptic architecture in human pluripotent stem cell model. Stem Cell Rep. *18*, 1854–1869.

Yang, C.P., Gilley, J.A., Zhang, G., and Kernie, S.G. (2011). ApoE is required for maintenance of the dentate gyrus neural progenitor pool. Development *138*, 4351–4362. https://doi.org/10.1242/dev.065540.

Yuzwa, S.A., Borrett, M.J., Innes, B.T., Voronova, A., Ketela, T., Kaplan, D.R., Bader, G.D., and Miller, F.D. (2017). Developmental Emergence of Adult Neural Stem Cells as Revealed by Single-Cell Transcriptional Profiling. Cell Rep. *21*, 3970–3986. https://doi.org/10.1016/j.celrep.2017.12.017.

Zhang, L., Han, Z., He, H., Zhang, X., Zhang, M., Li, B., and Wu, Q. (2023). Meg8-DMR as the Secondary Regulatory Region Regulates the Expression of MicroRNAs While It Does Not Affect Embryonic Development in Mice. Genes *14*, 1264. https://doi.org/10.3390/genes14061264.

Zhao, J., Fu, Y., Yamazaki, Y., Ren, Y., Davis, M.D., Liu, C.C., Lu, W., Wang, X., Chen, K., Cherukuri, Y., et al. (2020). *APOE4* exacerbates synapse loss and neurodegeneration in Alzheimer's disease patient iPSC-derived cerebral organoids. Nat. Commun. *11*, 5540. https://doi.org/10.1038/s41467-020-19264-0.

Zhao, J., Ikezu, T.C., Lu, W., Macyczko, J.R., Li, Y., Lewis-Tuffin, L.J., Martens, Y.A., Ren, Y., Zhu, Y., Asmann, Y.W., et al. (2023). APOE deficiency impacts neural differentiation and cholesterol biosynthesis in human iPSC-derived cerebral organoids. Stem Cell Res. Ther. *14*, 214. https://doi.org/10.1186/s13287-023-03444-y.

Zheng, L.J., Su, Y.Y., Wang, Y.F., Schoepf, U.J., Varga-Szemes, A., Pannell, J., Liang, X., Zheng, G., Lu, G.M., Yang, G.F., and Zhang, L.J. (2018). Different Hippocampus Functional Connectivity Patterns in Healthy Young Adults with Mutations of APP/Presenilin-1/2 and APOEε4. Mol. Neurobiol. *55*, 3439–3450. https://doi.org/10.1007/s12035-017-0540-4.

Stem Cell Reports, Volume *20*

Supplemental Information

APOE4 impacts cortical neurodevelopment and alters network formation in human brain organoids

Karina K. Meyer-Acosta, Eva Diaz-Guerra, Parul Varma, Adyasha Aruk, Sara Mirsadeghi, Aranis Muniz-Perez, Yousef Rafati, Ali Hosseini, Vanesa Nieto-Estevez, Michele Giugliano, Christopher Navara, and Jenny Hsieh

Figure S1 - iPSC characterization

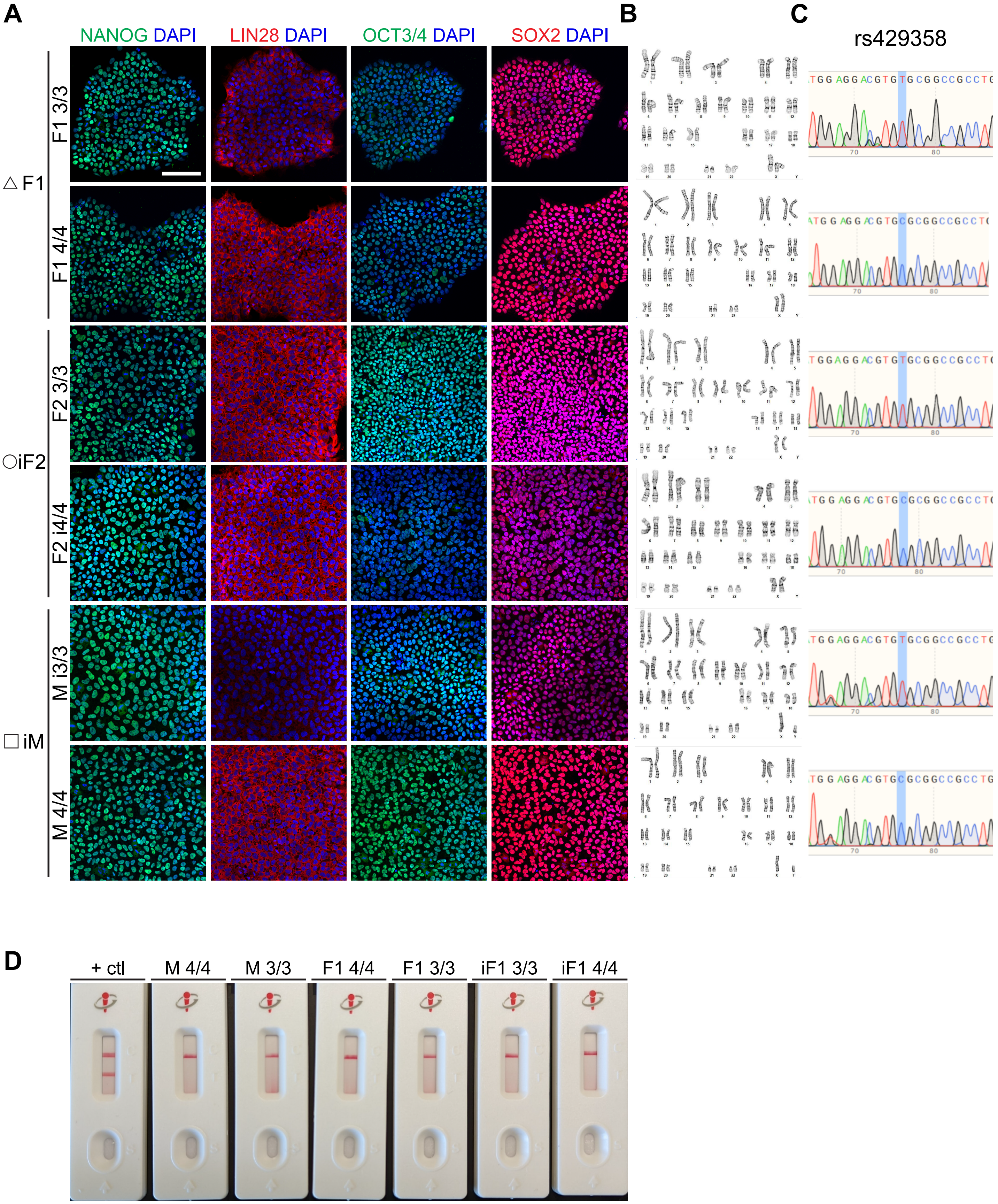

Figure S1. iPSCs characterization.
A) ICC representative images of each iPSC line used in this study showing the expression of the pluripotency markers Nanog, Lin28, OCT3/4, and SOX2. The nucleus was stained with DAPI. Each line was confirmed to have B) a normal karyotype, and C) a correct genotype. D) representative image of routine mycoplasma testing negativity, which are performed prior to and routinely during organoid culture. Scale bar = 100 μm.

Figure S2 - CO 1

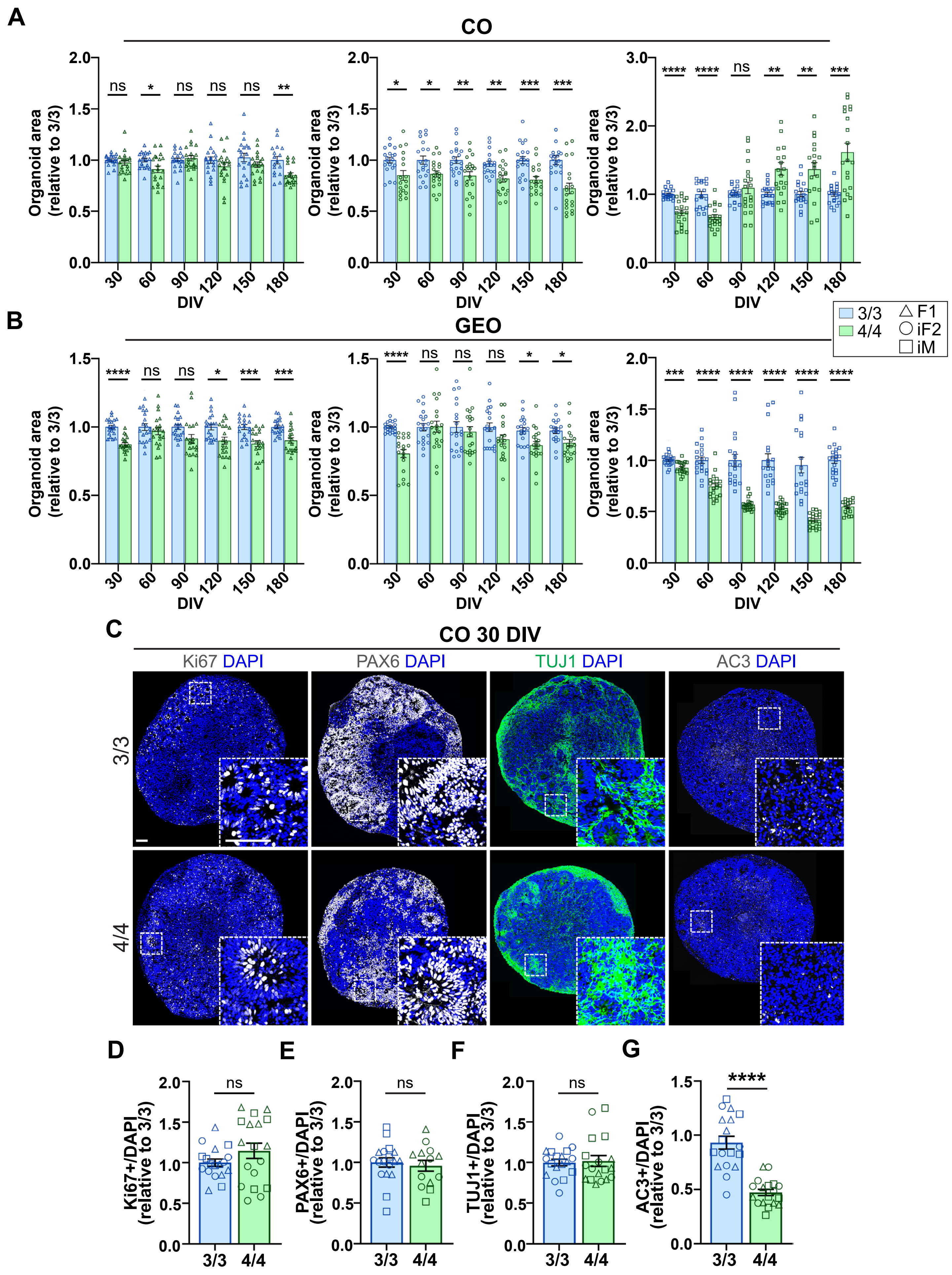

Figure S2. Size and characterization of COs at early time points (Related to Figure 2).
Assessment of organoid size of COs and GEOs across 30-180 DIV and IHC analysis of cellular phenotypes for COs at 30 DIV. (A-B) Organoid area across DIV, represented relative to APOE3/3 for: A) COs and B) GEOs, separated by iPSC line pair. C) Representative IHC images of APOE3/3 and APOE4/4 COs immunostained for Ki67, PAX6, TUJ1, and AC3. (D-G) Quantification of marker-positive cells over DAPI, represented relative to APOE3/3: D) Ki67 E) PAX6 F) TUJ1 and G) AC3. Organoid area: N = 57-60 organoids. IHC: N = 16-18 organoids. Data are presented as mean ± SEM, from 3 *APOE3/3* and 3 *APOE4/4* iPSC lines (2 replicates per iPSC line). Unpaired t-tests with Welch's correction were used to determine significance. Scale bar = 100 μm.

Figure S3 - CO 2

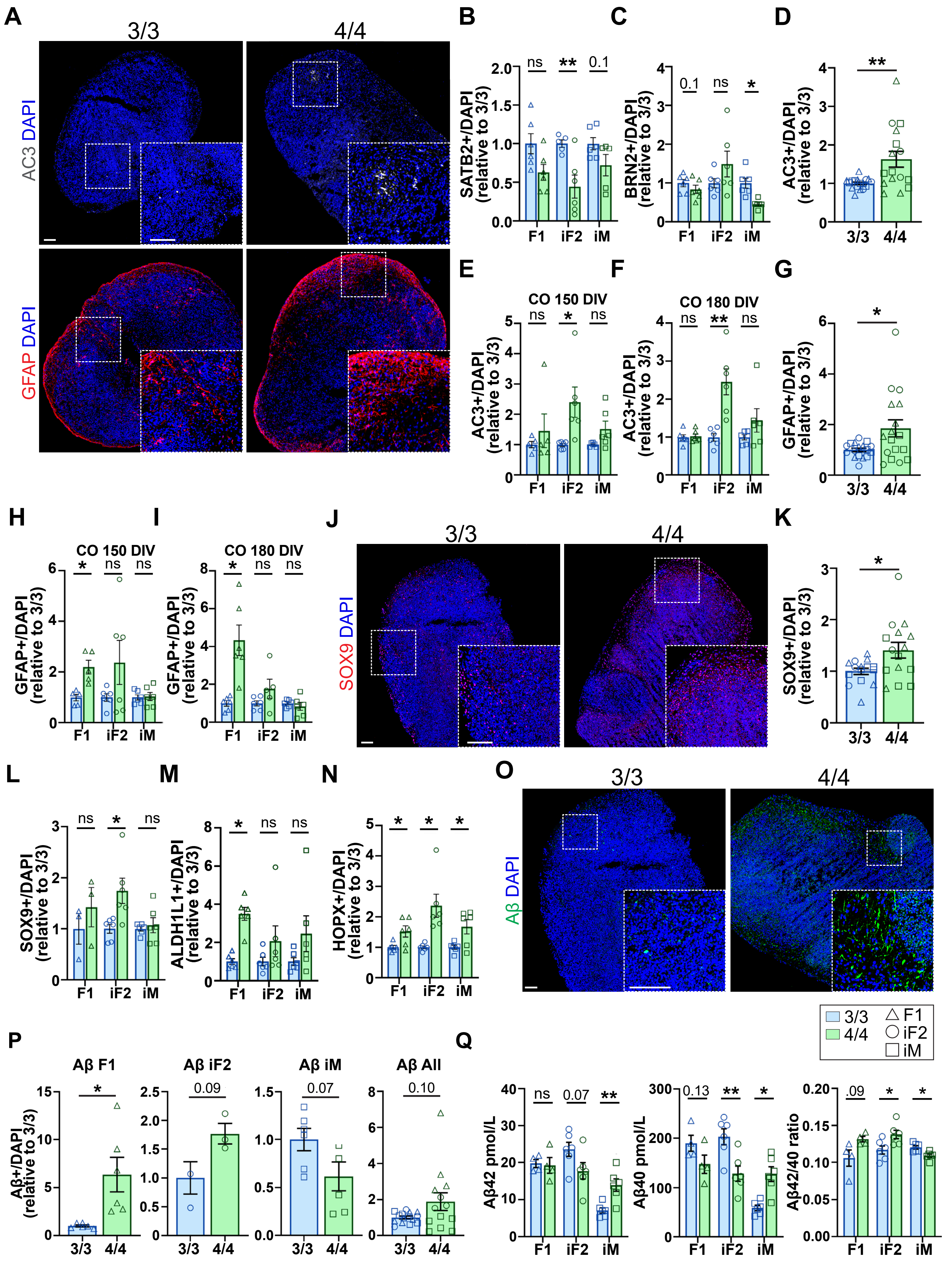

Figure S3. Characterization of COs at gliogenic time points (Related to Figure 2).
Characterization of COs at 150 and 180 DIV to evaluate cell type-specific markers, cell death, and Aβ pathologies via IHC and ELISA. A) Representative IHC images of *APOE3/3* and *APOE4/4* COs immunostained for AC3 and GFAP at 150 DIV. (B-C) Cortical neuron marker analysis corresponding to Figure 2, separated by iPSC line: B) SATB2 and C) BRN2. (D-F) AC3 quantification at 150 and 180 DIV: D) Combined analysis at 150 DIV. E) 150 DIV, separated by line. F) 180 DIV, separated by line. (G-I) GFAP IHC at 150 and 180 DIV: G) Combined analysis at 150 DIV. H) 150 DIV, separated by line. I) 180 DIV, separated by line corresponding to Figure 2. (J-L) SOX9 IHC at 180 DIV: J) Representative images of SOX9 in *APOE3/3* and *APOE4/4* COs. K) Combined analysis. L) Separated by line. (M-N) IHC analysis of other glial markers separated by line corresponding to Figure 2: M) ALDH1L1 and N) HOPX. O) Representative IHC images of Aβ (D54D2 antibody) in COs at 180 DIV. P) Aβ IHC quantification separated by iPSC line and combined. Q) ELISA from media of secreted Aβ42 and Aβ40 levels and Aβ42/Aβ40 ratio separated by line in COs at 180 DIV. Data are represented as mean ± SEM from 3 *APOE3/3* and 3 *APOE4/4* iPSC lines (2 replicates per iPSC line). IHC: N =16-18 organoids, 3 organoids per replicate. ELISA: N = 16 samples (each pooled from 3–4 organoids). Unpaired t-tests with Welch's correction were used to determine significance. * $p < 0.05$, ** $p < 0.01$, ns: not significant. Scale bar = 100 μm.

Figure S4 - GEO

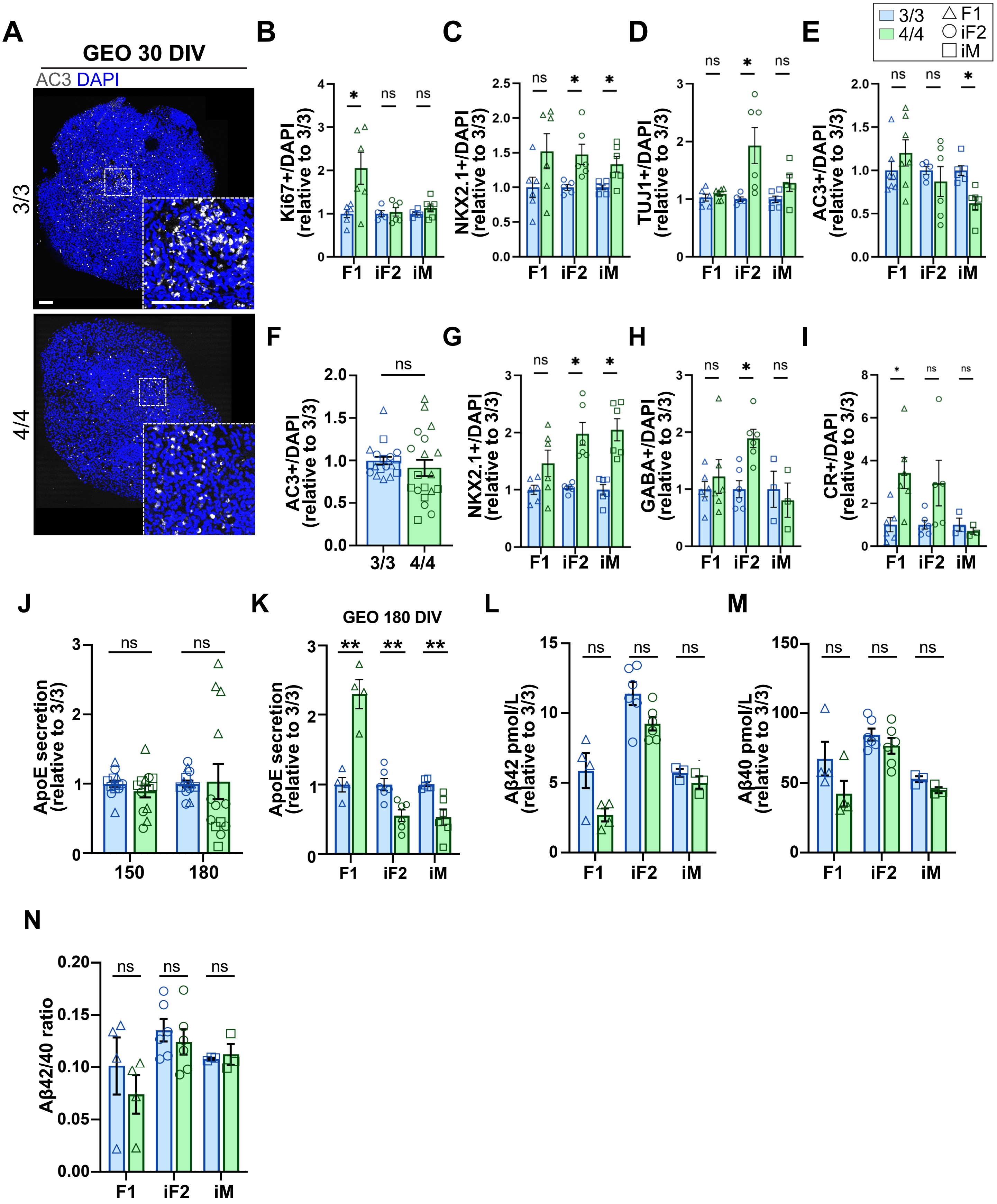

Figure S4. Characterization of GEOs (Related to Figure 3).

Characterization of GEOs for cell death at early (30 DIV) and secreted protein levels at late (150 and 180 DIV) time points. Includes additional IHC graphs corresponding to Figure 3. **A)** Representative IHC images of AC3 in *APOE3/3* and *APOE4/4* GEOs at 30 DIV. **(B-D)** IHC quantification at 30 DIV, separated by iPSC line: **B)** Ki67, **C)** NKX2.1, and **D)** TUJ1 corresponding to Figure 3. **(E-F)** AC3 IHC analysis at 30 DIV: **E)** separated by line and **F)** combined analysis. **(J-K)** ApoE secretion from GEOs measured by ELISA: **J)** Combined analysis at 150 and 180 DIV. **K)** 180 DIV, separated by line. Data shown relative to *APOE3/3*. **(L-N)** Aβ secretion in GEOs at 180 DIV measured by ELISA: **L)** Aβ42, and **M)** Aβ40, and **N)** Aβ42/Aβ40 ratio, separated by line. IHC: N =16-18 organoids (3 *APOE3/3* and 3 *APOE4/4* iPSC lines). ELISA: 1–3 samples per iPSC line, each with 2 replicates. Data are represented relative to APOE3/3 and as mean ± SEM. Unpaired t-tests with Welch's correction were used to determine significance. * $p < 0.05$, ** $p < 0.01$, ns: not significant. Scale bar = 100 μm.

Figure S5 - RNAseq

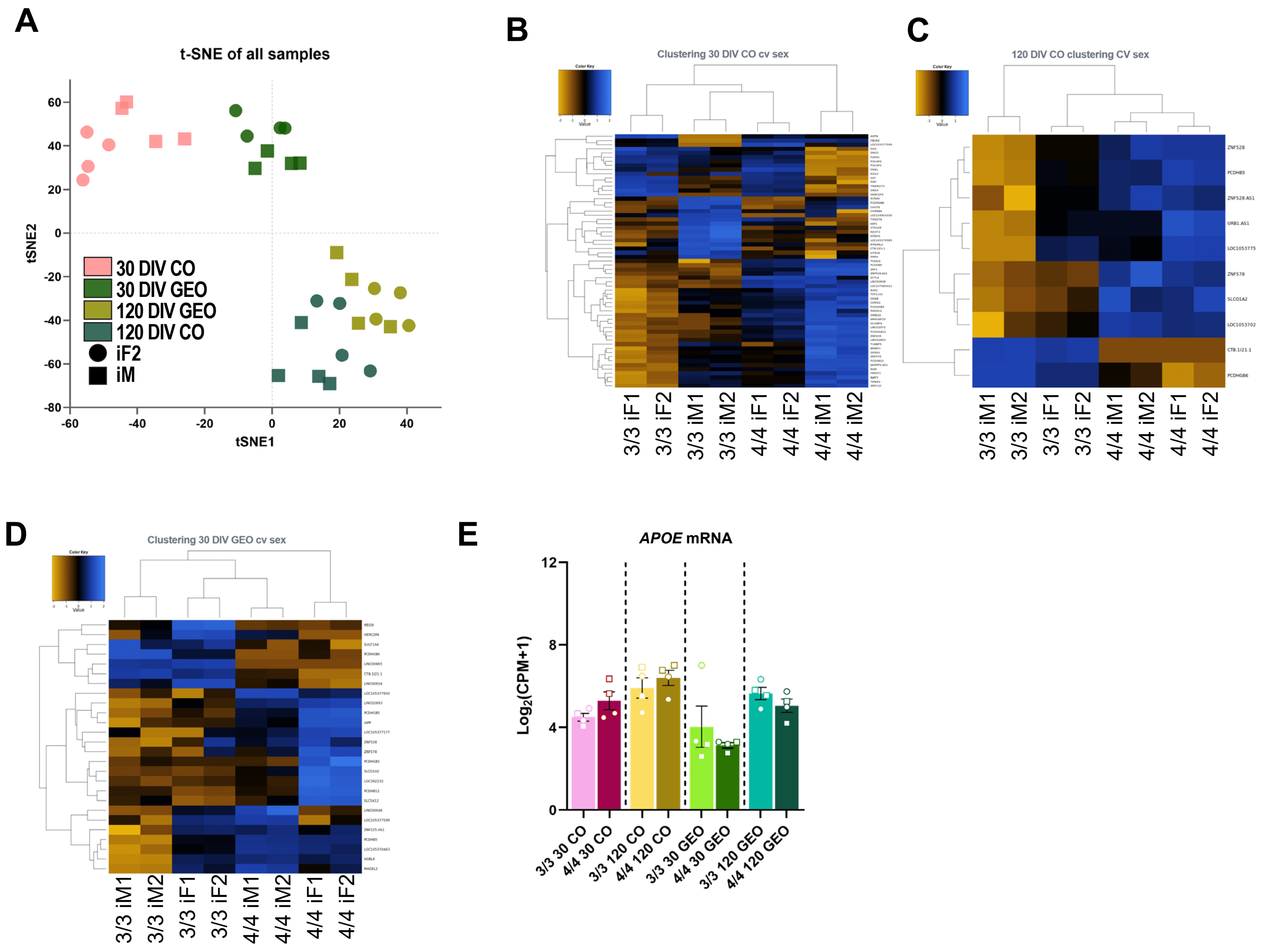

Figure S5. Bulk RNA-seq characterization of COs and GEOs (Related to Figure 4).
Bulk RNA sequencing of COs and GEOs derived from isogenic APOE3/3 and APOE4/4 pairs, collected at 30 and 120 DIV. **A)** t-SNE Scatter plot shows organoid clustering by type and timepoint. Points are colored by organoid type and timepoint. **(B-D)** Heatmap shows clustering analysis of each line and replicate for differentially abundant features in *APOE3/3* vs. *APOE4/4*: **B)** COs at 30 DIV, and **C)** COs at 120 DIV, and **D)** GEOs at 30 DIV. Dendrograms show Euclidean clustering. Data are CPM-normalized, log2-transformed, and z-score scaled. **E)** Barplot shows the $\log_2$(CPM+1) normalized values of *APOE* expression on the y-axis and all conditions on the x-axis. Bars are colored by condition and data is represented as mean ± SEM.

Figure S6 - MEA and gene expression

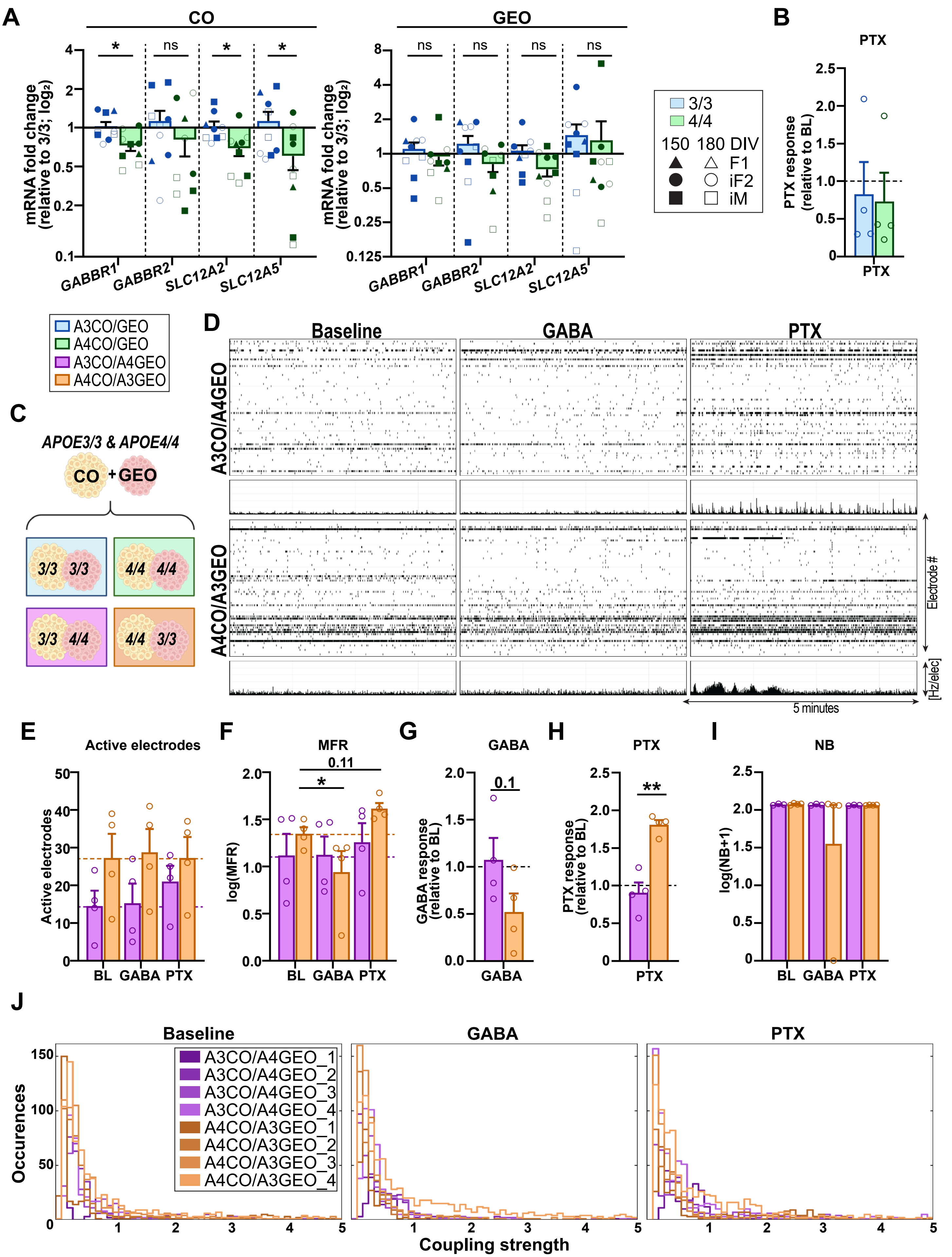

Figure S6. GABA-related gene expression and mixed assembloid MEA analysis (Related to Figure 5). Assessment of GABA-related gene expression at 150 and 180 DIV in COs and GEOs, and functional activity in mixed-genotype assembloids at 220 DIV. **A)** qRT-PCR analysis of $GABA_B$ receptors (*GABBR1*, *GABBR2*) and Cl^- transporters (*SLC12A2*, *SLC12A5*) in *APOE3/3* and *APOE4/4* COs and GEOs. N = 9 samples (3 *APOE3/3* and 3 *APOE4/4* iPSC lines, 2 replicates per line, 3 organoids per sample). **B)** PTX response for APOE3 and APOE4 matched assembloids, measured as the MFR ratio of PTX over BL for each respective assembloid. **C)** schematic of matched and mix-matched assembloid and corresponding barplot color. **(D-J)** MEA analysis of A3CO and A4CO mixed assembloids. **D)** Raster plots show spikes across 60 electrodes over a representative 5 minute recording during baseline, GABA, and PTX conditions. Mean firing rate (0–10 Hz/sec) is shown beneath each raster. **(E-I)** Bar plots show: **E)** active electrodes, **F)** MFR (log-transformed), **G)** GABA response, **H)** PTX response, **I)** total network bursts (log transformed: log(NB+1)) in each condition. **J)** Histograms of cross-correlation strength between electrode pairs (x-axis: coupling strength, y-axis: frequency). Only significant coupling peaks are shown. N = 4 assembloids per genotype from isogenic *APOE3/3* and *APOE4/4* iPSC lines. Data are represented as mean ± SEM. 2-way ANOVA with Sidak's multiple comparisons was used for bar plots; unpaired t-tests were used for other comparisons. * $p < 0.05$, ns: not significant.

Supplemental Tables

Table S1: List of primary antibodies used in this study				
**Antibody**	Host Species	Company	Cat. Number	Dilution
**Anti-AC3**	Rabbit	Cell Signaling	9661	1:400
**Anti-ALDH1L1**	Rabbit	Abcam	Ab177463	1:200
**Anti-BRN2**	Mouse	EMD Millipore	MABD51	1:50
**Anti-Calretinin**	Mouse	Swant	CR6B	1:1000
**Anti-D54D2 (Aβ)**	Rabbit	Cell Signaling	8243	1:500
**Anti-GABA**	Rabbit	Sigma	A2052	1:1000
**Anti-GFAP**	Chicken	Millipore Sigma	AB5541	1:1000
**Anti-HucHuD**	Mouse	Invitrogen	A-21271	1:500
**Anti-HOPX**	Rabbit	Sigma	HPA055888	1:500
**Anti-Ki67**	Mouse	BD	550609	1:500
**Anti-Ki67**	Rat	Invitrogen	14-5698-82	1:200
**Anti-LIN28**	Rabbit	Cell Signaling	3978	1:1000
**Anti-MAP2ab**	Mouse	Sigma	M1406	1:500
**Anti-NANOG**	Mouse	Thermoscientific	MA1-017	1:500
**Anti-NKX2.1**	Rabbit	Abcam	ab133737	1:500
**Anti-Oct3/4**	Mouse	Santa Cruz	sc-5279	1:1000
**Anti-PAX6**	Rabbit	Biolegend	901301	1:300
**Anti-SATB2**	Rabbit	Abcam	AB34735	1:500
**Anti-SOX2**	Rabbit	Millipore	AB5603	1:1000
**Anti-SOX9**	Goat	R&D Systems	AF3075	1:500
**Anti-TUJ1**	Mouse	Sigma	T8660	1:400

Table S2: List of primers for RT-qPCR, related to Figure S6		
**Gene**	Forward	Reverse
**GABBR1**	TTCAACTACAACAACCAGACCATTACCG	GCGTCCATGCCATCCGAGAG
**GABBR2**	CCCCTGCGAAGGACAGTGGAG	AACAACCGAACAACATGAGAAGTCCC
**GAPDH**	GGAAGCTTGTCATCAATGGAAATC	TCAGCAGAGGGGGCAGAGAT
**SLC12A2**	AACGCTGTTGCAGTTGCTATGTATGTG	AGATACCTAAAAGAATCACGACTGTAATGGC
**SLC12A5**	CTGCAGAACATCTTTGGCGTCATC	CAGCAGGCACAACACCATTCGT

**Table S3: Software and statistics, related to Figures 4 and 5**		
	Reference	URL
**nf-core-rnaseq (pipeline v3.12.0)**	(Ewels et al., 2020; Harshil Patel, 2024)	https://nf-co.re/rnaseq
**Trim Galore**	(FelixKrueger/TrimGalore)	https://www.bioinformatics.babraham.ac.uk/projects/trim_galore/
**STAR**	(Dobin et al., 2012)	10.1093/bioinformatics/bts635
**RSEM**	(Li and Dewey, 2011)	10.1186/1471-2105-12-323
**DESeq2**	(Love et al., 2014)	10.18129/B9.bioc.DESeq2
**fGSEA**	(Korotkevich et al., 2021)	http://bioconductor.org/packages/fgsea/
**MSigDB**	(Dolgalev, 2021; Liberzon et al., 2015; Subramanian et al., 2005)	https://www.gsea-msigdb.org/gsea/msigdb/human/genesets.jsp?collection=C8
**msigdb R**	(Dolgalev, 2021)	https://CRAN.R-project.org/package=msigdbr
**edgeR**	(Robinson et al., 2009)	10.18129/B9.bioc.edgeR
**Rtsne**	(Krijthe, 2015)	https://github.com/jkrijthe/Rtsne
**Pluto**	pluto.bio	https://pluto.bio
**ImageJ**	(Schneider et al., 2012)	https://imagej.nih.gov/ij/
**Matlab**	The MathWorks Inc., Natick, Massachusetts	https://www.mathworks.com
**Julia**	(Bezanson et al., 2017)	https://julialang.org/
**QSpikeTools**	(Mahmud et al., 2014)	https://github.com/mgiugliano/SpiQ

Supplemental methods

iPSC characterization continued:

APOE4/4 female iPSC line (CW50129) was sourced from CIRM. An iPSC line was generated by the UTSA stem cell core from fibroblast samples (Coriell) using Cytotune-iPS Sendai Reprogramming kit (Thermofisher). We also confirmed no integration of reprogramming genes. All iPSCs were expanded to create stocks from 3-4 passages then karyotyped (Wicell), and experiments were performed within 5 passages of karyotype. For pluripotency, iPSC's were grown on coverslips in a 24-well plate and were fixed at 75% confluence for immunocytochemistry (see below). All iPSC lines were negative for mycoplasma throughout the study using either genomic PCR (Cat No. MP0035-1KT, Millipore sigma) or supernatant (Cat No. rep-mys-20, Invitrogen). iPSCs were maintained without antibiotics with no visible signs of microbial presence.

Detailed organoid generation protocol:

iPSCs were seeded in a 6-well plate at 275,000 cells per well. 12-24 hours prior to organoid generation, 1% DMSO was added during routine feeding to enhance the differentiation of iPSCs (Chetty et al., 2013). On day 0 of differentiation, iPSCs at 70% confluence were detached and dissociated with Accutase (Sigma-Aldrich) and washed with mTESR. iPSCs were seeded at 9,000 cells per well of an ultra-low attachment 96-well round bottom plate (Corning Cat. No. 7007) in mTeSR containing Y27632 (20 µM), and medium changes were performed daily. On Day 1, media was changed to neural induction medium TeSR™-E6 medium (Cat. No. 05946, Stemcell Technologies). On days 1-5, Dual-SMAD inhibition was performed using SMAD inhibitors dorsomorphin (2.5 µM, Sigma) and SB-431542 (10 µM, Tocris), and wnt-inhibitor XAV 939 (1.2 µM, Tocris) was added to enhance forebrain differentiation. On days 6-24, media was replaced with neural medium (Neurobasal A, B-27 without Vitamin A, Glutamax, penicillin/streptomycin) containing the growth factors, bFGF (20 ng/ml, Peprotech) and EGF (20 ng/ml, Peprotech). On day 15, organoids were transferred to a 24-well plate (Corning Cat. No. 3473) and were maintained on an orbital shaker (80 RPM) to promote oxygenation. On day 25, media was replaced with neural medium containing BDNF (20 ng/ml, Peprotech) and NT-3 (20 ng/ml, Peprotech) until day 42. For ganglionic eminence organoid generation, Wnt inhibitor IWP-2 (5 µM, Selleckchem) was added on days 4-22, and the SMO pathway activator, SAG (100 nM, Selleckchem) on days 12-22.

IHC and sample preparation

For iPSCs: iPSCs were plated on glass coverslips in a 24-well plate. When cells reached 60-70% confluence, they were fixed with 4% paraformaldehyde (PFA) for 15 minutes and washed 3 times with PBS. Coverslips were stained using the same protocol as described below for organoids. **For organoids:** At each time point, organoids were harvested and fixed in 4% PFA for 1-2 hours at room temperature or overnight at 4 °C, then incubated at 4 °C in 30% sucrose until they sank (24-48 hours). Organoids are embedded in OCT compound and frozen on dry ice. Organoids were cryosectioned at 14-µm, in serial, on glass slides. For IHC, organoids were incubated in blocking buffer (1X Carbo-Free Blocking solution (Vector labs, Cat. No. SP-5040-125), 0.3% Triton X-100 in TBS) for 1 hour at room temperature (RT), and primary antibodies (See Table S1) overnight at 4 °C in a humidified chamber. After 3 TBS washes, slides were incubated with secondary antibodies (Jackson Immunoresearch, 1:400, or Alexafluor 488, 1:1000, or Alexafluor 647, 1:400) for 2 hours at RT. Slides were washed, 4',6-diamidino-2-phenylindole (DAPI; Sigma, Cat. No. D9542) was added to the second wash to label nuclei, then coverslipped using polyvinyl Alcohol solution (PVA; Sigma, Cat. No. BP168-122). **Confocal image processing for presentation:** To improve image presentation in figures, the brightness and contrast was adjusted using ImageJ fiji software. Adjustments were made equally in APOE3/3 and APOE4/4 for each marker.

Bulk-RNA sequencing details

75 paired-end RNAseq (2x75bp flowcell) was performed by the UTSA genomics core on all 32 samples at the same time with Element Biosciences AVITI DNA Sequencer, using the Freestyle Cloudbreak chemistry at a depth of 25-30 million reads per sample. Analysis performed and plots created using Pluto (https://pluto.bio). paired_end FASTQ files were processed using the nf-core-rnaseq pipeline (v3.12.0)(auto strandedness)(Ewels *et al.*, 2020). Adapter sequences were removed with Trim Galore. Reads were aligned with STAR (Dobin *et al.*, 2012) to GRCh38 (NCBI, p.14, release 110) and quantified to gene counts using RSEM (Li and Dewey, 2011). **Differential gene expression analysis:** Differential expression analysis was performed with the DESeq2 R package (Love *et al.*, 2014), which tests for differential expression based on a model using the negative binomial distribution. **Gene set enrichment analysis (GSEA):** was performed using the fgsea R package and the fgsea Multilevel() function (Korotkevich *et al.*, 2021). The $\log_2$ fold change from APOE3/3 and APOE4/4 differential expression comparisons were used to rank genes. C5: Gene Ontology gene sets - biological process gene set collection from the Molecular Signatures Database (MSigDB) (Dolgalev, 2021) was curated using the msigdbr R package(Liberzon *et al.*, 2015; Subramanian *et al.*, 2005). Prior to running GSEA, the list of gene sets was

filtered to include only gene sets with between 5 and 1000 genes. **Clustering analysis:** Clustering analysis was performed for each cell line and replicate using differentially abundant features in the comparison of *3/3* vs. *4/4* for each time point and organoid type. Features were filtered using an adjusted p-value ≤ 0.01 and $\log_2$ fold change threshold of 1 (showing features that are both positive and negative). Prior to plotting, the data was counts per million (CPM)-normalized, $\log_2$-transformed, and z score-transformed. **Venn diagram:** Overlap genes analysis was performed using the following gene sets: CO 30 DIV and GEO 30 DIV. For gene set CO 30 DIV, differential genes were defined as genes with an adjusted p-value of less than or equal to 0.05, and a fold change greater than 1.2. For gene set GEO 30 DIV, differential genes were defined as genes with an adjusted p-value of less than or equal to 0.05, and a fold change greater than 1.2. **APOE CPM bar plot:** Bar plot showing the counts per million (CPM)-normalized values for APOE on the y-axis and *3/3* and *4/4* for each time point and organoid type on the x-axis. CPM values were calculated using the cpm() function in the edgeR R package (Robinson *et al.*, 2009) with log=F. Bars are drawn at the average value for each Group and are colored according to Group. Error bars represent the average +/- standard error of the mean (SEM). Values on the y-axis are displayed on a symmetrical $\log_{10}$ scale. **t-distributed stochastic neighbor embedding analysis (t-SNE):** Barnes-Hut t-Distributed Stochastic Neighbor Embedding (t-SNE)(Laurens van der Maaten; 2014) was performed by applying the Rtsne() R function (Krijthe, 2015) to raw values for all 43,236 targets in the experiment and all samples. An initial principal components analysis (PCA) step was run prior to t-SNE. The perplexity parameter used was 10.3.

Detailed MEA recording and analysis:

All recordings were carried out inside a humid (i.e., 95% R.H.) incubator at 37℃ and 5% CO2. Organoids were maintained using BrainPhys^{TM} hPSC Neuron kit (05795; STEMCELL Technology) for a week prior to recording session. For each session, individual organoids were placed on 3D – MEA (60-3DMEA200/12/80iR-Ti, Multichannel system, Harvard Bioscience). To ensure optimal attachment to the MEA surface, organoids were immersed in 200 µL of BrainPhys^{TM}. Sequential recordings were performed as follows: baseline, 20 µM GABA (56-12-2, Sigma-Aldrich), and 58 µM picrotoxin (124-87-8, Sigma-Aldrich), for 30 minutes each condition, with recording started 10 minutes after drug application. Fluorinated Teflon thin film (MEA-MEM-set5, Multichannel systems, Harvard Bioscience) was used to seal the chip, to block evaporation and contamination. Raw electrical potentials were amplified using electronic amplifier (ME2100-Mini, Multichannel systems, Harvard Bioscience), sampled at 25 kHz/channel, and digitized at 16-bit resolution. Data were stored on disk via software (Experimenter, Multichannel systems, Harvard Bioscience). To detect the time of occurrence of putative action potentials (i.e. spike times), a peak detection algorithm with adaptive threshold was employed, for spontaneous network-wide synchronization of spike times (i.e. network bursts) could be detected and quantified (Mahmud *et al.*, 2014; Quiroga et al., 2004). The events were visualized as a raster plot and further analyzed by spike train analysis (Mahmud *et al.*, 2014; Quiroga *et al.*, 2004). Interaction between electrode pairs was derived using cross-correlation analysis of spike times (Knox, 1981), limited to inter-spike delays less than 500 ms and quantified by 3 ms bins. Each cross correlogram was divided by the square root of spike number in a spike train to account for firing rate modulation. The peak value from the cross-correlogram represented connectivity strength of the electrode pairs. Peak value distribution across experimental conditions were generated to facilitate a comparison of coupling strength. To assess the significance of these peaks (coupling strength), their corresponding inter-spike intervals (ISI) were randomly shuffled to achieve surrogate spike times with identical distribution of ISI. The peaks were considered significant if they showed values larger than the mean plus 3 standard deviations of the cross-correlogram of the surrogate.

Additional Statistics

To determine whether *APOE4/4* lines were significantly different from one another for each IHC marker, statistics were performed between all markers separated by line using a Brown-Forsythe and Welch’s one-way ANOVA. If either test was significant, we calculated the p-value for each *APOE4/4* comparison using Dunnett’s T3 correction for multiple comparisons.

Supplemental References

(FelixKrueger/TrimGalore).

Bezanson, J., Edelman, A., Karpinski, S., and Shah, V.B. (2017). Julia: A Fresh Approach to Numerical Computing. SIAM Review 59, 65-98. 10.1137/141000671.

Chetty, S., Pagliuca, F.W., Honore, C., Kweudjeu, A., Rezania, A., and Melton, D.A. (2013). A simple tool to improve pluripotent stem cell differentiation. Nat Methods 10, 553-556. 10.1038/nmeth.2442.

Dobin, A., Davis, C.A., Schlesinger, F., Drenkow, J., Zaleski, C., Jha, S., Batut, P., Chaisson, M., and Gingeras, T.R. (2012). STAR: ultrafast universal RNA-seq aligner. Bioinformatics 29, 15-21. 10.1093/bioinformatics/bts635.

Dolgalev, I. (2021). msigdbr: MSigDB Gene Sets for Multiple Organisms in a Tidy Data Format. .

Ewels, P.A., Peltzer, A., Fillinger, S., Patel, H., Alneberg, J., Wilm, A., Garcia, M.U., Di Tommaso, P., and Nahnsen, S. (2020). The nf-core framework for community-curated bioinformatics pipelines. Nature Biotechnology 38, 276-278. 10.1038/s41587-020-0439-x.

Harshil Patel, P.E., Jonathan Manning, Maxime U Garcia, Alexander Peltzer, Rickard Hammarén, Olga Botvinnik, Adam Talbot, Gregor Sturm, nf-core bot, Matthias Zepper, Denis Moreno, Pranathi Vemuri, Mahesh Binzer-Panchal, Ezra Greenberg, silviamorins, Lorena Pantano, Robert Syme, Gavin Kelly, Friederike Hanssen, James A. Fellows Yates, Jose Espinosa-Carrasco, rfenouil, Luke Zappia, Chris Cheshire, Edmund Miller, marchoeppner, Peng Zhou, Sarah Guinchard, Gisela Gabernet (2024). nf-core/rnaseq (latest version).

Knox, C.K. (1981). Detection of neuronal interactions using correlation analysis. Trends in Neurosciences 4, 222-225. https://doi.org/10.1016/0166-2236(81)90070-9.

Korotkevich, G., Sukhov, V., Budin, N., Shpak, B., Artyomov, M.N., and Sergushichev, A. (2021). Fast gene set enrichment analysis. bioRxiv, 060012. 10.1101/060012.

Krijthe, J.H. (2015). Rtsne: T-Distributed Stochastic Neighbor Embedding using Barnes-Hut Implementation.

Laurens van der Maaten, G.H. Visualizing Data using t-SNE. Journal of Machine Learning Research 9, 2579-2605, 86.

Laurens van der Maaten, G.H. (2014). Accelerating t-SNE using Tree-Based Algorithms. Journal of Machine Learning Research 15, 3221-3245, 93.

Li, B., and Dewey, C.N. (2011). RSEM: accurate transcript quantification from RNA-Seq data with or without a reference genome. BMC Bioinformatics 12, 323. 10.1186/1471-2105-12-323.

Liberzon, A., Birger, C., Thorvaldsdóttir, H., Ghandi, M., Mesirov, J.P., and Tamayo, P. (2015). The Molecular Signatures Database (MSigDB) hallmark gene set collection. Cell Syst 1, 417-425. 10.1016/j.cels.2015.12.004.

Love, M.I., Huber, W., and Anders, S. (2014). Moderated estimation of fold change and dispersion for RNA-seq data with DESeq2. Genome Biology 15, 550. 10.1186/s13059-014-0550-8.

Mahmud, M., Pulizzi, R., Vasilaki, E., and Giugliano, M. (2014). QSpike tools: a generic framework for parallel batch preprocessing of extracellular neuronal signals recorded by substrate microelectrode arrays. Frontiers in Neuroinformatics 8. 10.3389/fninf.2014.00026.

Quiroga, R.Q., Nadasdy, Z., and Ben-Shaul, Y. (2004). Unsupervised spike detection and sorting with wavelets and superparamagnetic clustering. Neural Comput 16, 1661-1687. 10.1162/089976604774201631.

Robinson, M.D., McCarthy, D.J., and Smyth, G.K. (2009). edgeR: a Bioconductor package for differential expression analysis of digital gene expression data. Bioinformatics 26, 139-140. 10.1093/bioinformatics/btp616.

Schneider, C.A., Rasband, W.S., and Eliceiri, K.W. (2012). NIH Image to ImageJ: 25 years of image analysis. Nature Methods 9, 671-675. 10.1038/nmeth.2089.

Subramanian, A., Tamayo, P., Mootha, V.K., Mukherjee, S., Ebert, B.L., Gillette, M.A., Paulovich, A., Pomeroy, S.L., Golub, T.R., Lander, E.S., and Mesirov, J.P. (2005). Gene set enrichment analysis: A knowledge-based approach for interpreting genome-wide expression profiles. Proceedings of the National Academy of Sciences 102, 15545-15550. doi:10.1073/pnas.0506580102.
